# Understanding adaptations in a community-vetted COVID-19 testing program

**DOI:** 10.3389/frhs.2025.1408940

**Published:** 2025-04-07

**Authors:** Breanna J. Reyes, Stephenie Tinoco Calvillo, Angel Lomeli, Arleth A. Escoto, Maria Linda Burola, Kelli L. Cain, Linda Salgin, Maria Balbuena-Bojorquez, Anne-Marie Engler, Marva Seifert, Louise C. Laurent, Nicole A. Stadnick, Borsika A. Rabin

**Affiliations:** ^1^ Department of Obstetrics, Gynecology, and Reproductive Sciences, University of California San Diego, La Jolla, CA, United States; ^2^ Herbert Wertheim School of Public Health and Human Longevity Science, University of California San Diego, La Jolla, CA, United States; ^3^ Department of Research and Health Promotion, San Ysidro Health, San Ysidro, CA, United States; ^4^ Joint Doctoral Program in Public Health, San Diego State University/University of California San Diego, San Diego, CA, United States; ^5^ Department of Medicine, University of California San Diego, La Jolla, CA, United States; ^6^ Department of Psychiatry, University of California San Diego, La Jolla, CA, United States; ^7^ Altman Clinical and Translational Research Institute, Dissemination and Implementation Science Center, University of California San Diego, La Jolla, CA, United States; ^8^ Child and Adolescent Services Research Center, San Diego, CA, United States

**Keywords:** adaptation, COVID-19 testing, border community, community-based, implementation science

## Abstract

**Background:**

Adaptations are expected when complex public health interventions are implemented in dynamically and rapidly changing real-world settings, as seen for many programs during the COVID-19 pandemic. Systematic documentation of adaptations to intervention components and strategies are critical when assessing their impact on implementation. Here, we report processes used for tracking and evaluating adaptations made during the CO-CREATE project, which aimed to address COVID-19 testing disparities in the San Ysidro US/Mexico border community.

**Methods:**

The study utilized a longitudinal, prospective, mixed methods approach to systematically document and assess adaptations across the pre-implementation, early and mid/late-implementation phases of the project. Aggregated from a combination of sources (i.e., meeting notes, Advisory Board transcripts, and periodic reflections), adaptations were entered weekly into an electronic database that captured information on 16 characteristics and were validated by study staff. The impacts of the adaptations were determined using a team consensus approach and based on the outcomes from the Reach, Effectiveness, Adoption, Implementation, and Maintenance framework. Each adaptation was evaluated to determine whether it increased, decreased, had no effect, or not applicable to the RE-AIM outcomes. Data were analyzed using descriptive statistics.

**Results:**

98 adaptations were identified, and most were identified by research staff (*n* = 79, 75.2%). Planned adaptations were defined as those discussed between at least two research team members prior to implementation. Unplanned adaptations were defined as a change made without shared discussion and agreement among at least 2 research team members. Most adaptations were planned (*n* = 93, 94.9%). Of those that were planned, (*n* = 21, 22.6%) occurred during pre-implementation, (*n* = 26, 28.0%) during early implementation, and (*n* = 46, 49.4%) during mid/late implementation. Of those that were unplanned, (*n* = 1, 20.0%) occurred during pre-implementation and (*n* = 4, 80.0%) occurred during implementation. Most adaptations (*n* = 45, 45.9%) had a positive impact (i.e., increase) on the efficiency of delivery of services, meaningful engagement of partners, and reach of community members through the program.

**Conclusion:**

This work describes our systematic and prospective approach to document and analyze adaptations over a two-year period and assesses the impact of these adaptations. Lessons learned from this work can be used to develop best practices for adapting interventions to ensure sustainable implementation and address disparities in public health and clinical programs.

## Introduction

Adaptations are planned or unplanned changes made to a public health or clinical program and related implementation strategy or context that occur before, during, and after implementation. These changes are commonly made to increase the alignment of the program and/or implementation strategy with the complex and dynamic real-world implementation context and as a result to enhance the success of the program in specific settings and for specific populations (1–4). Adaptations also serve to prevent program inequalities by ensuring that they meet the needs of disadvantaged groups (5). These changes should be informed by or in some cases initiated by community members or local leaders to incorporate multiple perspectives, needs, and priorities (6). Although there are frameworks available to guide the adaptation process (4, 7), as well as to support adaptation documentation and reporting (8–10), the essential elements to be documented and how to capture the impact of adaptations on implementation and effectiveness outcomes have not been rigorously established (3, 4, 7, 11) A key gap in the literature is the analysis of the impact of adaptations on the adoption, implementation, and sustainment of interventions (1, 12). There is also a need to understand best practices for modifying interventions to achieve sustainable implementation and address gaps (4, 8, 10).

While adaptations are expected in various types of research studies, community-based research programs that were implementing solutions for challenges that arose from the COVID-19 pandemic required a larger number and more substantial program adjustments to respond to the rapidly changing context and meet the emerging needs of health systems and communities (13). Community-driven Optimization of COVID-19 testing to Reach and Engage underserved Areas for Testing Equity- in Women and Children (CO-CREATE) was a two-year project aimed at decreasing COVID-19 disparities experienced by underserved communities by offering no-cost testing and preventive care services. This partnership between UC San Diego, San Ysidro Health [a Federally Qualified Health Center (FQHC) in South San Diego], and the Global Action Research Center, allowed for the development of a community guided approach to increasing COVID-19 testing in the San Ysidro community, a highly impacted area in the San Diego County (14, 15). The CO-CREATE project was funded by the National Institutes of Health within the RADx-UP Initiative.

In this paper, we describe our longitudinal prospective documentation of adaptations over this two-year CO-CREATE project. This includes real-time tracking of adaptations made to meet community needs and the project's response to COVID-19 pandemic related milestones. We also discuss lessons learned from documenting and understanding the impact of these adaptations.

## Methods

We used a theoretically driven, longitudinal, prospective, mixed methods approach to systematically document the adaptations made during the pre-implementation phase, early implementation phase, and implementation phases for the CO-CREATE project. We then summarized and analyzed these data to provide an overview of the different adaptations, and our process to assess their impact on project implementation. We evaluated the impact of the adaptation on the RE-AIM outcomes. These variables were specifically operationalized for this study and included type and number of community members engaged or reached (Reach), increased community testing or other relevant outcomes (Effectiveness), participation of teams or staff in CO-CREATE implementation (Adoption), consistent delivery of quality of care or costs (Implementation), maintenance or sustainability of community participation/impact (Maintenance), reimbursement or financial implications for the practice (Implementation), efficiency, and meaningful engagement.

### The Co-CREATE program

The CO-CREATE study was designed and implemented using a community-driven approach (14–16). The project consisted of two aims. The first aim focused on establishing a Community and Scientific Advisory Board (CSAB) with the intention of co-creating a COVID-19 testing approach that incorporates community needs, scientific evidence, and the realities and priorities of San Ysidro Health. The CSAB guided the process for the co-creation and implementation of the project, and also made recommendations for project-wide adaptations. The CSAB was composed of 22 members representing community residents, public health researchers, and clinical partners (16). A combination of a Theory of Change process and Appreciative Inquiry were undertaken by the CSAB to guide these processes (16).

The second aim focused on the implementation, iterative refinement, and evaluation of the COVID-19 testing program. The CO-CREATE study focused on providing no-cost COVID-19 testing and inviting participants to enroll in the research arm of the study by completing a questionnaire. Participation was open to FQHC patients and the surrounding community, the Mexico United States border community of San Ysidro. Individuals could return for repeat testing as frequently as twice a week regardless of their participation in the study's research arm.

To improve access to testing, all on-site staff were bilingual (English/Spanish), and all participant-facing materials were provided in both languages. Our testing site offered a COVID-19 polymerase chain reaction (PCR) test, with a walk-up no-cost option offered during usual clinic hours. The minimal demographic data required for reporting to the County of San Diego Health and Human Services Agency (i.e., name, date of birth, address, and phone number) and preferred method of contact were collected prior to testing. Test results were returned to individuals within 1–3 days of sample collection. If the result was positive, a clinical provider contacted the participant by phone to provide further instructions on isolation recommendations and to answer any COVID-19-related questions; these calls were made in either English or Spanish, with interpreters being available to clinical providers who were not fluent in Spanish.

If individuals elected to enroll in the research arm of the study, they were consented by study staff and asked to complete the questionnaire that captured participants behaviors and beliefs surrounding COVID-19, testing and vaccine related experiences, and demographic and health related data (15). Study participants who completed the survey were compensated $20 USD for completing the initial questionnaire and $10 USD for completing an abbreviated questionnaire at follow-up. Community members of all ages were eligible to participate except for those who were unable to provide assent or consent (e.g., those with severe developmental delays or disabilities) or did not have a legal guardian who could consent on their behalf.

**Figure 1:**
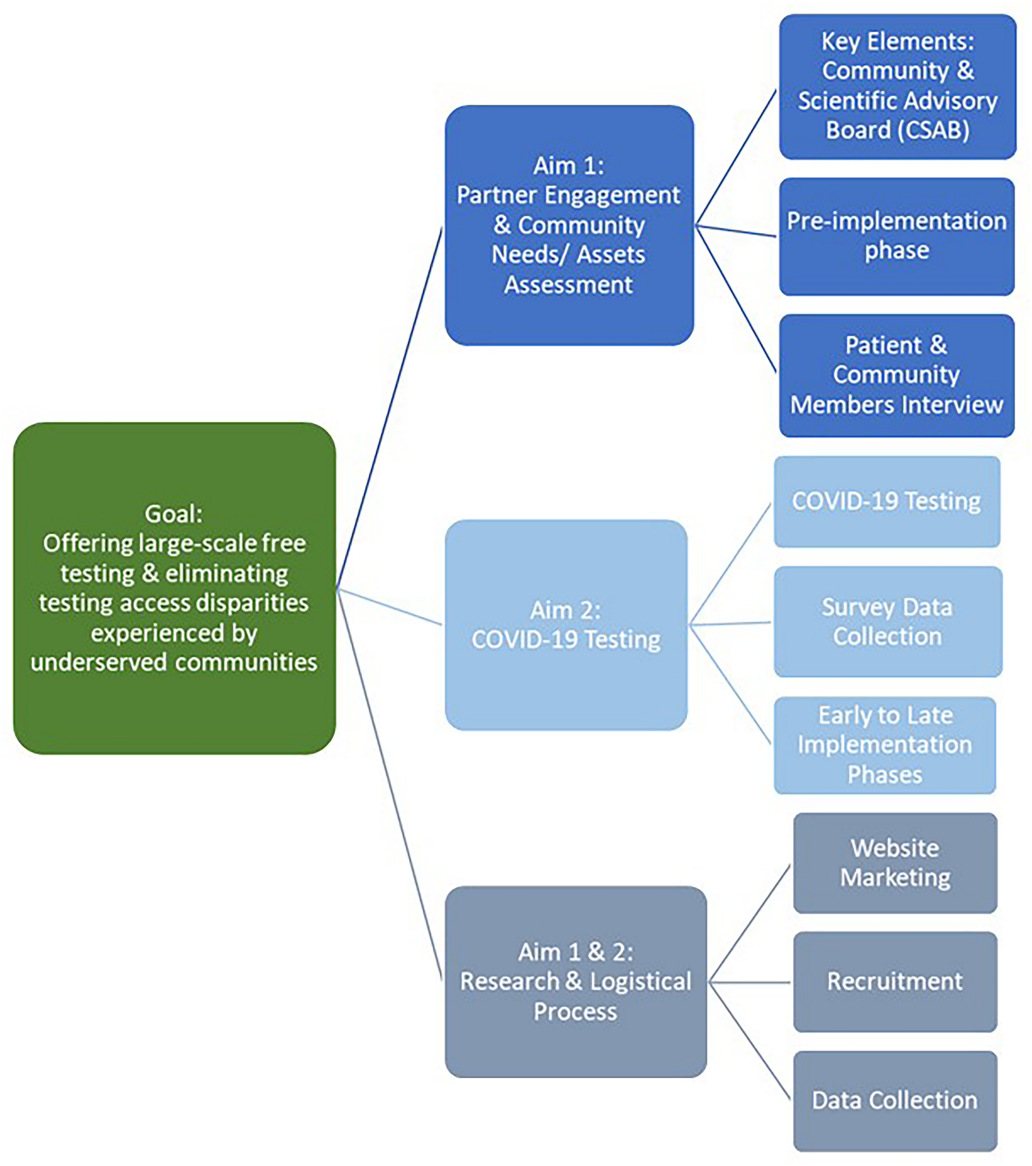
Project Key elements by aims.

Figure 1 A flowchart displaying the overarching goal of the CO-CREATE program, the Specific Aims, and key elements of each Aim over the pre-implementation, and implementation phases. More information about the CO-CREATE project is available in Lomeli et al., Rabin et al., Reyes et al., Salgin et al., and Stadnick et al.

### Setting

CO-CREATE was implemented at a single COVID-19 testing site located at a Federally Qualified Health Center on the US-Mexico border in San Diego County. This FQHC is the region's second largest FQHC, serving vulnerable populations across the San Diego County and rooted in providing affordable health care and social services to patients of all ages (17).

### Data collection and sources for adaptations

We used the Framework for Reporting Adaptations and Modifications to Evidence-based interventions (FRAME) (8, 10) and supplemented it with the reach, effectiveness, adoption, implementation, and maintenance (RE-AIM) framework to assess intent and impact of adaptations (18, 10). The overall approach was modeled after work by Hall et al. (19) and the multi-method protocol described by Rabin et al. (20–22). Briefly, FRAME was developed to guide systematic documentation of adaptations while RE-AIM can provide an expansion to FRAME to guide the documentation of intent (Why and adaptation was made) and impact (what was the outcome of the adaptation).

We documented adaptations across both Aims with some adaptations being associated with both Aims. An Adaptation Team was assembled to guide the documentation and analysis process. The Adaptation Team included on-site study coordinators, program managers, student researchers, and implementation researchers, with two study coordinators designated as the lead Adaptation Documentation Specialists.

Adaptations were documented using four methods: (1) review of notes from weekly research, clinical, and community partner meetings; (2) CSAB transcripts; (3) bi-weekly email reminders sent to the research and clinical team; and (4) periodic reflections with research staff, clinical partners, and community partners (20). While most adaptations were documented concurrently for the first few months of the project, we reviewed meeting notes retrospectively to capture adaptations that happened before the adaptation documentation system was launched. Once the adaptations system was in place, adaptations were captured in real time. A trained clinical research coordinator with support from the Adaptation Team entered all adaptations into a central database. To identify additional adaptations, an email was sent bi-weekly to the entire study team asking them to send a short description of any adaptations they had observed. Adaptations received via email were then reviewed by the lead coordinator who followed up with the source for any additional information about the adaptation, if necessary. Finally, periodic reflections were conducted with the research and clinical team and the community partner on a quarterly basis to review the list of adaptations and add any additional adaptations. Periodic reflections were 20–30 min guided discussions that encouraged the clinical team and community partner to reflect on implementation events that may have led to an adaptation (23). This allowed the adaptation to capture adaptations that may have been missed, and it helped assess the impact of the adaptation.

Although there are data entry fields and methods to create an adaptation database, adaptations for this project were added to a CO-CREATE Adaptation database that was modeled after databases from prior studies systematically documenting adaptations (2–3). Database fields were adapted to reflect the specific characteristics of the CO-CREATE project. We documented 11 characteristics of each adaptation which mapped onto FRAME and RE-AIM dimensions. The 11 adaptation characteristics are summarized in Table 1.

**Table 1:** Adaptation characteristics.

Adaptation characteristics	Explanation
Name and description of adaptation	The adaptation title and a summary of what was changed. Brief description of the adaptation that was made (try to keep it to 1–2 sentences but provide enough context that it stands alone. For example: recruitment criteria were changed to include all patients with XX code as well.
Date an adaptation was implemented	The timeframe when the adaptation occurred (i.e., pre-implementation, early-implementation, implementation).
Core component changed	Aim 1: Partner Engagement and Community Needs/Assets Assessment, Aim 2: COVID-19 Testing Aim 1 & 2: Research and Logistical Process)
Stages of the COVID-19 pandemic associated with the adaptation	Delta B.1.617.2 s surge, Omicron BA.1 Omicron BA.2 Vaccine/booster availability
Planned vs. unplanned^a^	Planned if changes that were made after thoughtful discussion with at least one team member to address an issue.
Elements changed	Setting Format Personnel involved Target population How components are operationalized
Type of change	Tailoring to individuals Adding a component Removing a component, condensing a component Extending a component, changing order of components Repeating a component Integrating with other programs
Who was responsible for the change	Entire or most of team, Clinical Research Coordinator (CRC) Other researcher –CSAB Principle or Co-Co-Principal Investigator
How or on what basis was the change made	Based on our vision or values Based on our framework Based on our knowledge or experience working with patients Based on financial incentives Based on feedback or suggestions
Why was the change made	Reach Effectiveness Adoption Implementation Maintenance Response to external pressures or policy
Was this adaptation a result of external factors or internal issues	External factors: related to non-research team processes Internal issues: related to research team processes
Short term impact of the adaptation	Reach: number or type of patients engaged/reached Effectiveness: Increase community testing or other outcome Adoption: participation of teams or staff Implementation: consistent delivery of quality of care or costs Maintenance: maintenance or sustainability of community participation

^a^ Indicates diversion from the definition proposed by Stirman and colleagues (8, 10).

Supplementary Table S1 provides the full data collection instrument. Throughout the data collection, weekly virtual meetings were held for the Adaptation team to review all adaptations for accuracy and completeness.

### Data management and analysis

**Figure 2:**
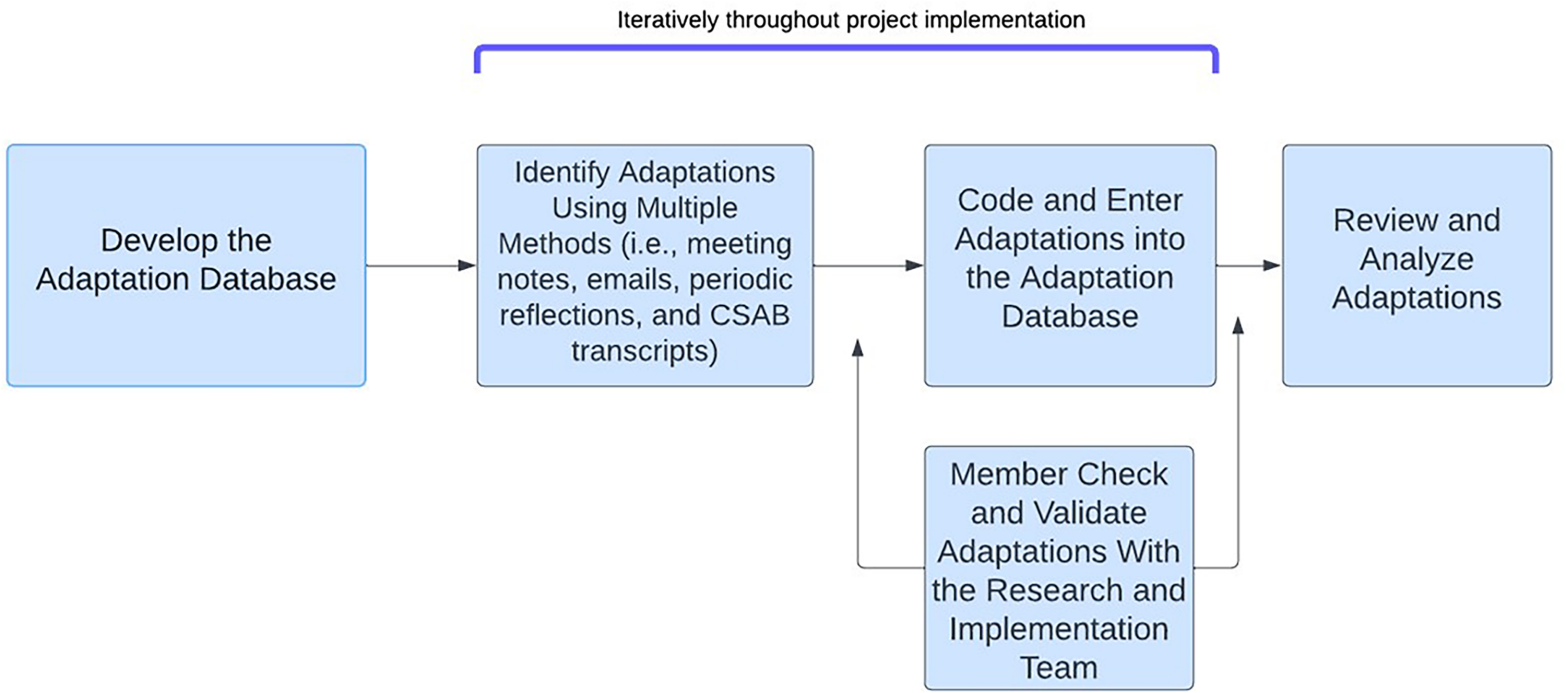
Process for identifying CO-CREATE adaptations.

Figure 2 demonstrates the data sources and analytical process. Once all adaptations were entered into the database, the Adaptation Team identified all unique adaptations (i.e., combined those that were entered as a duplicate from different sources) and evaluated the short-term impact of the changes to key outcomes that mapped on the RE-AIM framework outcomes (i.e., reach, effectiveness, adoption, Implementation, maintenance). After data cleaning and impact assessment were completed, the adaptation data was validated by the broader study team familiar with the intervention and the occurred adaptations. During this process, the team also identified the adaptation's perceived impact on a set of key RE-AIM outcomes aligned with key elements of implementation and effectiveness using a team-based consensus approach. Impact variables included type and number of community members engaged or reached (Reach), increased community testing or other relevant outcomes (Effectiveness), participation of teams or staff in CO-CREATE implementation (Adoption), consistent delivery of quality of care or costs (Implementation), maintenance or sustainability of community participation/impact (Maintenance), reimbursement or financial implications for the practice (Implementation), efficiency, and meaningful engagement.

For each adaptation, the Adaptation Team discussed each potential impact variable and decided if the adaptation resulted in an increase, decrease, no change or if the given impact outcome was not relevant for the adaptation. When the Adaptation team did not have enough information to make this decision, we engaged additional members of the research and implementation team.

Adaptation data were then organized by the key adaptation characteristics. We also looked at whether differences in characteristics emerged when comparing adaptations that were made pre-implementation (i.e., start of study through before COVID-19 testing started), early implementation (i.e., first three months of testing), and implementation and late implementation (i.e., combined to reflect adaptations starting the fourth month of testing through the end of data documentation).

Figure 2 Displays the process the CO-CREATE team used to determine adaptations throughout the project.

## Results

### Overview of adaptations

**Figure 3:**
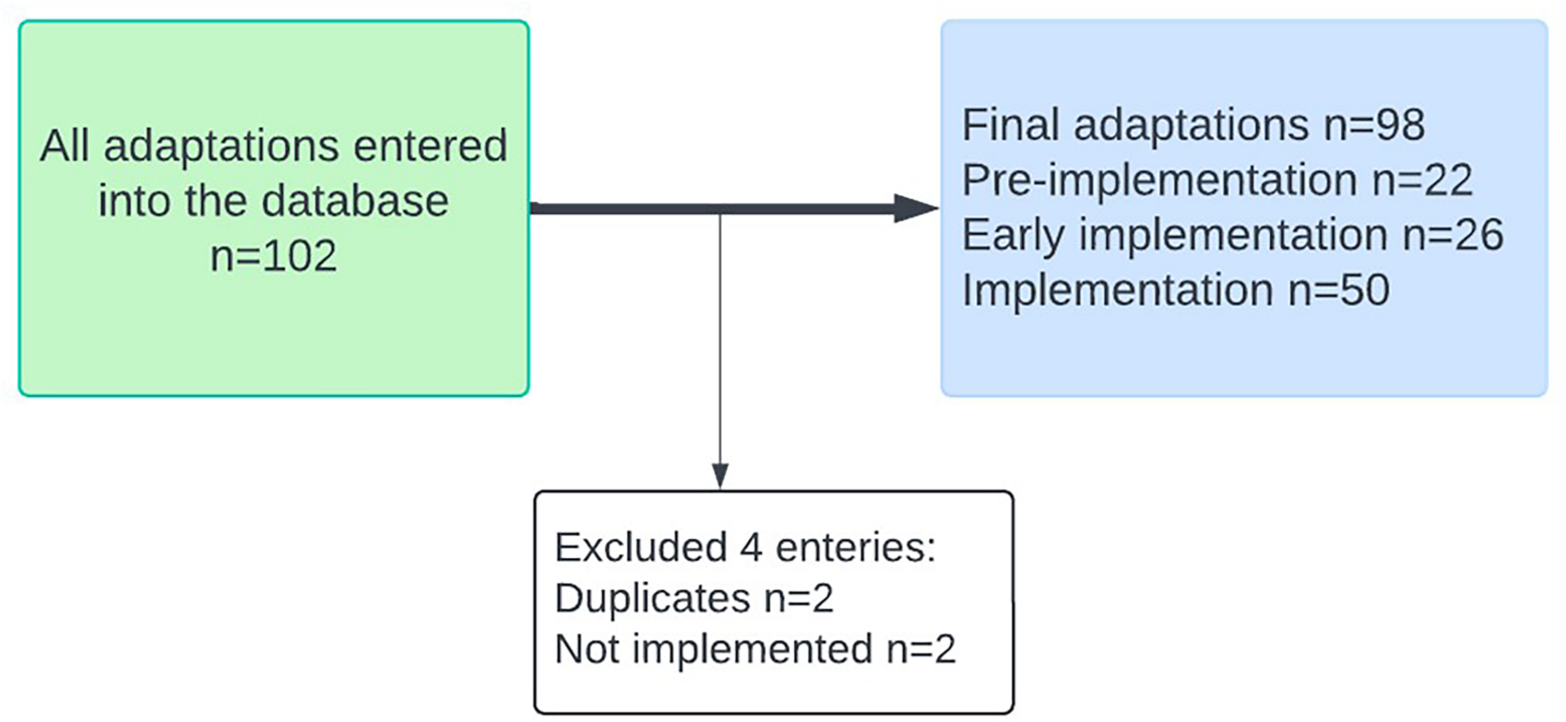
Unique adaptations for CO-CREATE.

A total of 102 adaptations were documented. After removing duplicates and entries representing proposed changes that were not implemented, 98 unique adaptations were included in this analysis, as seen in Figure 3. Supplementary Table S2 provides all adaptations tracked throughout the project.

### Timing of adaptations

Adaptations were documented at different time points during the study. Most adaptations were made in the implementation phase (*n* = 50, 51.0%) followed by the pre-implementation (*n* = 22, 22.4%), and the early implementation phases (*n* = 26, 26.5%).

### Source

Adaptations were identified through a review of old and concurrent meeting notes reviewed by University of California San Diego (UCSD) staff researchers to capture adaptations throughout the project. Most adaptations regardless of implementation stage were initiated by UCSD research staff (*n* = 79, 75.2%). During pre-implementation, 12 adaptations (15.2%) were introduced by UCSD research staff. During Early implementation (*n* = 20, 25.3%) and implementation (*n* = 47, 59.5%) most adaptations were initiated by a UCSD research staff. Adaptations were not initiated by all groups, as seen in Table 2, Federally Qualified Health Center investigators and providers did not initiate any adaptations.

**Table 2:** Unique adaptations identified across time points and adaptation constructs.

Adaptation constructs	Pre-implementation	Early implementation	Implementation	Total
Source				
University of California San Diego Research Staff	12 (15.2%)	20 (25.3%)	47 (59.5%)	79
University of California San Diego Investigator(s)	2 (22.2%)	5 (55.6%)	2 (22.2%)	9
Community and Scientific Advisory Board	10 (90.9%)	1 (9.1%)	0 (0%)	11
Federally Qualified Health Center Research Staff	1 (20.0%)	3 (60.0%)	1 (20.0%)	5
Federally Qualified Health Center Investigator(s)	0 (0.0%)	0 (0.0%)	0 (0.0%)	0
Federally Qualified Health Center Provider(s)	0 (0.0%)	0 (0.0%)	0 (0.0%)	0
Other	0 (0.0%)	1 (100.0%)	0 (0.0%)	1
Core locations & functions				
Maternal Child Health Center	6 (18.2%)	16 (48.5%)	11 (33.3%)	33
San Ysidro Health Center	0 (0.0%)	0 (0.0%)	34 (100.0%)	34
Community Sites	6 (28.6%)	11 (52.4%)	4 (19.0%)	21
Community and Scientific Advisory Board	9 (81.8%)	0 (0.0%)	2 (18.2%)	11
Across project	1 (33.3%)	0 (0.0%)	2 (66.7%)	3
Planned vs. unplanned				
Planned	21 (22.6%)	26 (28.0%)	46 (49.4%)	93
Unplanned	1 (20.0%)	0 (0.0%)	4 (80.0%)	5
Elements changed				
Setting	6 (37.5%)	2 (12.5%)	8 (50.0%)	16
Format	8 (26.7%)	6 (20.0%)	16 (53.3%)	30
Personnel Involved	2 (18.2%)	3 (27.3%)	6 (54.5%)	11
Target Population	0 (0.0%)	3 (60.0%)	2 (40.0%)	5
How the intervention/program is presented/delivered	12 (26.0%)	17 (37.0%)	17 (37.0%)	46
Other	1 (14.3%)	2 (28.6%)	4 (57.1%)	7
Type of change				
Tailoring to individuals	7 (26.9%)	3 (11.5%)	16 (61.5%)	26
Adding a component	7 (20.6%)	6 (17.6%)	21 (61.8%)	34
Removing a component	1 (16.7%)	2 (33.3%)	3 (50.0%)	6
Condensing a component	4 (50.0%)	3 (37.5%)	1 (12.5%)	8
Extending a component	6 (28.6%)	9 (42.8%)	6 (28.6%)	21
Substituting a component	4 (28.6%)	4 (28.6%)	6 (42.8%)	14
Changing the order of components	0 (0.0%)	3 (75.0%)	1 (25.0%)	4
Integrating with other programs we are doing	0 (0.0%)	0 (0.0%)	1 (100.0%)	1
Other	0 (0.0%)	0 (0.0%)	3 (100.0%)	3
Which core component is this change related to				
AIM 1: Partner Engagement and Community Needs/Assets Assessment	22 (91.7%)	0 (0.0%)	2 (8.3%)	24
AIM 2: COVID-19 Testing	0 (0.0%)	24 (36.3%)	44 (64.7%)	68
AIM 1 & AIM 2: Research and Logistical Process	1 (16.7%)	2 (33.3%)	3 (50.0%)	6
Other	0 (0.0%)	0 (0.0%)	2 (100.0%)	2
Who was responsible for the change				
Entire or most of team	2 (33.3%)	4 (14.3%)	22 (78.6%)	28
Clinical Research Coordinator (CRC)	9 (19.1%)	17 (36.2%)	21 (44.7%)	47
Community and Scientific Advisory Board	10 (90.9%)	1 (9.1%)	0 (0.0%)	11
Principle or Co-Principle Investigator(s)	2 (22.2%)	2 (22.2%)	5 (55.6%)	9
Federally Qualified Health Center Research Staff	1 (6.2%)	2 (12.5%)	13 (81.3%)	16
Federally Qualified Health Center Investigator(s)	0 (0.0%)	0 (0.0%)	2 (100.0%)	2
Federally Qualified Health Center Administration/Organization Leaders	1 (33.3%)	2 (66.7%)	0 (0.0%)	3
Other	0 (0.0%)	4 (66.7%)	2 (33.3%)	6
How or on what basis was the change made				
Based on our vision or values	2 (25.0%)	0 (0.0%)	6 (75.0%)	8
Based on our framework	0 (0.0%)	0 (0.0%)	0 (0.0%)	0
Based on our knowledge or experience of working with patients	6 (21.4%)	10 (35.7%)	12 (42.9%)	28
Based on QI data, summary information or results	0 (0.0%)	3 (60.0%)	2 (40.0%)	5
Based on pragmatic/practical considerations	3 (6.3%)	16 (33.3%)	29 (60.4%)	48
Based on financial incentives or payment	3 (27.3%)	2 (18.2%)	6 (54.5%)	11
Based on feedback or suggestions	18 (33.3%)	15 (27.8%)	21 (38.9%)	54
Other	6 (66.7%)	1 (11.1%)	2 (22.2%)	9
Why was the change made				
To increase the number or type of patients contacted (reach)	7 (20.0%)	13 (37.1%)	15 (42.9%)	35
To enhance the impact or success of the intervention for all or important subgroups (effectiveness)	17 (29.8%)	16 (28.1%)	24 (42.1%)	57
To make it possible to involve more teams, team members or staff (adoption)	4 (26.7%)	5 (33.3%)	6 (40.0%)	15
To deliver intervention more consistently; better for practice, patient flow or EHR (implementation)	4 (12.5%)	12 (37.5%)	16 (50.0%)	32
For practical reasons (implementation)	4 (11.8%)	17 (50.0%)	13 (38.2%)	34
To institutionalize or sustain the intervention (maintenance)	0 (0.0%)	2 (15.4%)	11 (84.6%)	13
To respond to external pressures or policy	4 (25.0%)	4 (25.0%)	8 (50.0%)	16
To save money or other resources (implementation)	3 (75.0%)	0 (0.0%)	1 (25.0%)	4
Other	0 (0.0%)	1 (20.0%)	4 (80.0%)	5
Was this adaptation a result of external factors or internal issues				
External	11 (27.5%)	3 (7.50%)	26 (65.0%)	40
Internal	11 (19.0%)	23 (39.7%)	24 (41.3%)	58

### Core locations & functions

The core locations and functions of the program included the two clinic sites [i.e., Maternal Children Health Center (MCHC) and San Ysidro Health Main Clinic (SYHC)], the community sites and the CSAB. Some adaptations were relevant to all locations (i.e., MCHC, SYHC, community sites). We documented (*n* = 6, 18.2%) pre-implementation changes, (*n* = 16, 48.5%) early implementation, and (*n* = 11, 33.3%) implementation changes at MCHC. SYHC only had changes occur during the implementation stage of the project (*n* = 34, 100.0%) as it was the location for CO-CREATE's walk up testing from August 2021 to October 2022. Adaptation patterns for the mobile community services (i.e., community sites) were similar to the pattern observed in the MCHC site. There were three adaptations that affected all three locations, and 11 adaptations were documented from the Community and Scientific Advisory Board (CSAB).

### Planned vs. unplanned

Most adaptations were planned (*n* = 93, 94.9%). Of those that were planned, 21 (22.6%) occurred during pre-implementation, (*n* = 26) 28.0% happened during early implementation, and (*n* = 46), 49.4% occurred during implementation. Of those that were unplanned (*n* = 1) 20.0% occurred during pre-implementation and (*n* = 4) 80.0% occurred during implementation.

Planned adaptations were defined as those discussed with at least two other team members using available/emerging information/data. Unplanned adaptations were defined as a change made without shared discussion and agreement among at least 2 research members. An example of an unplanned adaptation occurred during the project's pre-implementation phase, when the UCSD research staff experienced push back from the university when ordering gift cards provided to research participants who completed a survey. Modifications were made to the project's financial system, resulting in various issues related to project funding codes, project number, and approval processes being incorrect. This caused a delay in the project Clinical Research Coordinator (CRC) being able to order gift cards and essentially providing compensation (gift cards) to participants. An example of a planned adaptation was exploring different areas within the San Ysidro community to expand access to testing and have the option to test at different locations not just the FQHC. This adaptation was initiated by the Clinical Research Coordinator during the project's early implementation to increase the number and diversity of research participants. Although this adaptation was never implemented, UCSD research staff discussed with community members and university officials to properly meet mobile testing implementation requirements.

### Elements changed

Most adaptations were categorized as “How the intervention/program is presented/delivered” (*n* = 46, 42.6%). Many of these adaptations occurred during the early implementation (*n* = 17, 37.0%) and implementation (*n* = 17, 37.0%) phase of CO-CREATE. This was followed by “Format” (*n* = 30, 27.8%) and “Setting” (*n* = 16, 14.8%). Many of the adaptations that were in the “Format” category occurred during the pre-implementation (*n* = 8, 26.7%) and implementation (*n* = 16, 54.4%) phases of the project. Adaptations within the “Setting” category occurred during the pre-implementation (*n* = 6, 37.5%) and implementation (*n* = 8, 50.0%) phases.

### Type of change

Most adaptations fell under, “Adding a component” category (*n* = 34, 29.1%), with most of the adaptations occurring during the implementation phase (*n* = 21, 61.8%). This second most common category was “Tailoring to individuals” (*n* = 26, 22.2%), and like the category “Adding a component” most of the adaptations happened during the implementation phase (*n* = 16, 61.5%). Adaptations were also commonly seen in the “Extending a component” (*n* = 21, 17.9%) category with most of the adaptations occurring during the early implementation phase (*n* = 9, 42.8%).

### Core component associations

There were 24 (24.0%) adaptations related to Aim 1 elements and activities and most of these adaptations were associated with changes to the CSAB. Most adaptations regarding Aim 1 elements took place during the pre-implementation stage (*n* = 22, 91.7%). A total of 68 (68.0%) Aim 2 adaptations were documented with most documented regarding the following sub-element in Aim 2: COVID-19 testing. Most Aim 2-related adaptations took place in the implementation stage (*n* = 44, 64.7%).

### Who was responsible for the change

The CRC was responsible for most of the changes that occurred throughout the project (*n* = 47, 38.5%). Most of the adaptations made by the CRC occurred during the implementation stage (*n* = 21, 44.7%). The changes the CRC made were related to COVID-19 testing and recruitment. Most of the research team was responsible for 28 adaptations. Most happened during the implementation stage (*n* = 22, 78.6%), which consisted of COVID-19 testing, data collection, research and logistical processes.

### How or on what basis was the change made

CO-CREATE adaptations were made based “on feedback or suggestions” (*n* = 54), “pragmatic or practical considerations” (*n* = 48), and “on our knowledge or experience of working with patients” (*n* = 28). For all three of these categories, most adaptations occurred during the implementation stage of the project. During the implementation stage, adaptations based “on feedback or suggestions” accounted for (*n* = 21) 38.9%, “pragmatic or practical considerations” accounted for (*n* = 29) 60.4%, and those based “on our knowledge or experience of working with patients” accounted for (*n* = 12) 42.9% adaptations. It is also important to note that 11 of our adaptations were made based on financial incentives or payment. Most of these adaptations occurred during the implementation stage (*n* = 6) 54.5%. The rest occurred during the pre-implantation stage (*n* = 3) 27.3% and early implementation stage (*n* = 2) 18.2%. Throughout the project, none of the adaptations identified were based on our theoretical framework.

### Why was the change made

Changes that occurred during the CO-CREATE project were made to “enhance the impact or success of the intervention for all or important subgroups (effectiveness) (*n* = 57, 27.0%). The adaptations that were identified to fall under this category mainly happened during the implementation stage (*n* = 24, 42.1%). Changes were also made to “increase the number or type of patients contacted (reach) (*n* = 35, 16.6%). The adaptations that were identified to fall under this category mainly happened during the implementation stage (*n* = 15, 42.9%). Although there were a few changes that were made to save money or other resources (*n* = 4, 0.02%), the majority did occur during the pre-implementation stage (*n* = 3, 75.0%).

### Was this adaptation a result of external factors or internal issues

Adaptations were identified as being an external factor (*n* = 40, 40.8%) or an internal issue (*n* = 58, 59.2%) throughout different time points in the study (i.e., pre-implementation, early-implementation and implementation phase). For those identified as external factors, most occurred in the implementation phase (*n* = 26, 65.0%) followed by the pre-implementation (*n* = 11,27.5%), and the early implementation phase (*n* = 3, 7.50%). For those identified as internal issues, most occurred in the implementation phase (*n* = 24, 41.3%) followed by the pre-implementation (*n* = 11,19.0%), and the early implementation phase (*n* = 23, 39.7%).

### Distribution of adaptations across the COVID-19 pandemic milestones

**Figure 4:**
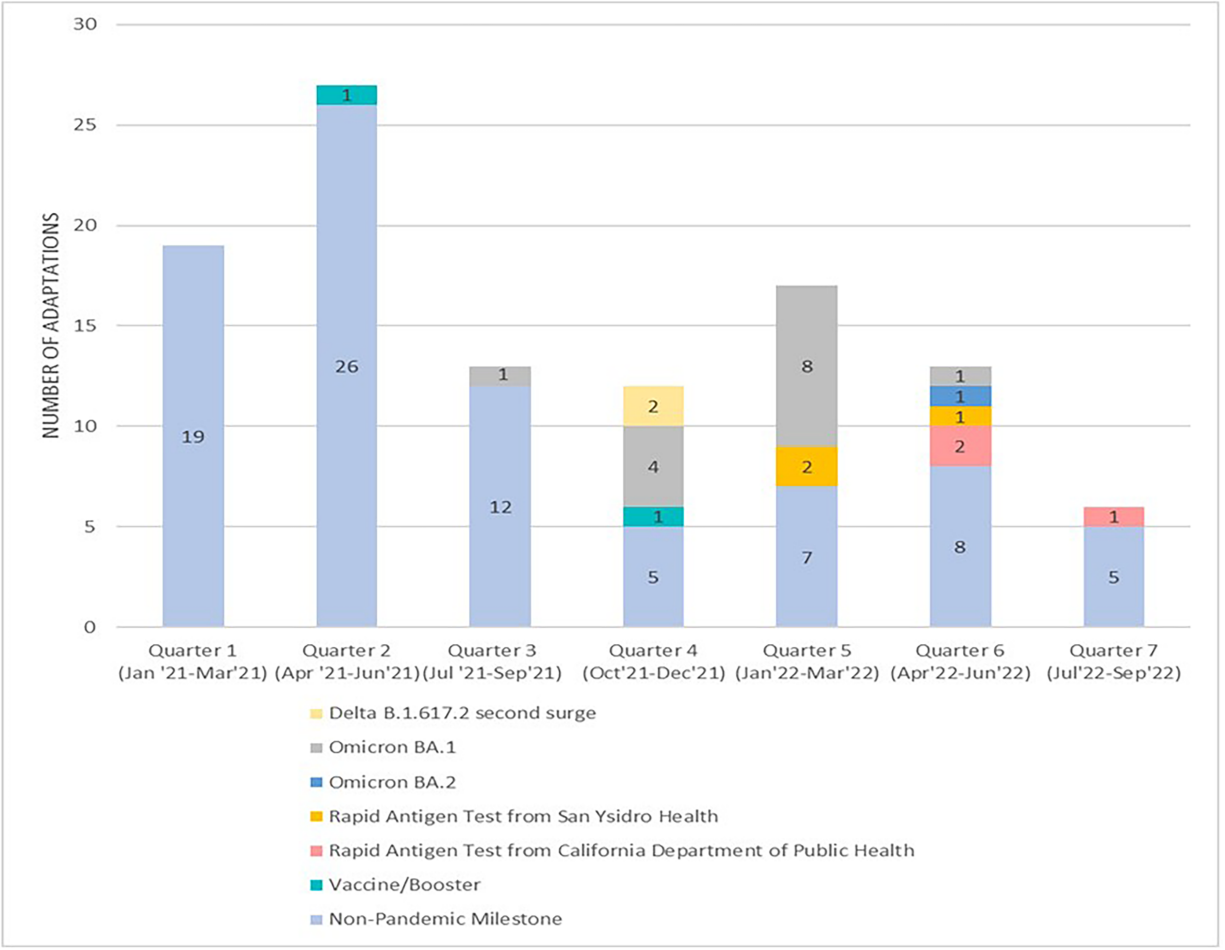
Adaptations organized by the COVID-19 pandemic timeline and related events and milestones.

Our team conducted a *post-hoc* analysis to map adaptations on the key events and milestones of the COVID-19 pandemic timeline. This was done to display the COVID-19 pandemic related stages associated with the project's adaptations. To achieve this, we identified key events and milestones associated with the COVID-19 pandemic that emerged during the implementation of our program. These key events and milestones included surges caused by various variants for the COVID-19 virus (i.e., Delta B.1.617.2 s surge, Omicron BA.1, Omicron BA.2) and the availability of rapid antigen tests from San Ysidro Health and the California Department of Public Health (CDPH) and the availability of the COVID-19 vaccine. We organized the timeline by seven quarters starting January 2021 through September 2022 which align with the timeline of our project when adaptations were documented. Adaptations associated with each event and milestone are color coded in Figure 4 and included in the order of occurrence changes in on-site safety protocols (Delta B.1.617.2 variant emergence), changes to testing site hours (Omicron BA.1 variant emergence), project start providing home-based rapid antigen tests (home rapid antigen tests via San Ysidro Health and then CDPH become available), change to return of results procedures (Omicron BA.2 variant emergence). Those associated with a non-pandemic milestone were related to research team personnel (i.e., hiring new staff and changing staff protocols to align with research aims), weather conditions, and making administrative changes (i.e., changing the time for team meeting, changing the type of incentives provided to research participants, and establishing partnerships with community organizations). During the project's early implementation period, from Quarter 1 to Quarter 3, very few adaptations (2 out of 59) were pandemic event or milestone related. Between Quarter 4 to Quarter 7, close to half of the adaptations (23 out of 48) were pandemic events or milestone related.

### Perceived impact of adaptations on RE-AIM outcomes

**Figure 5:**
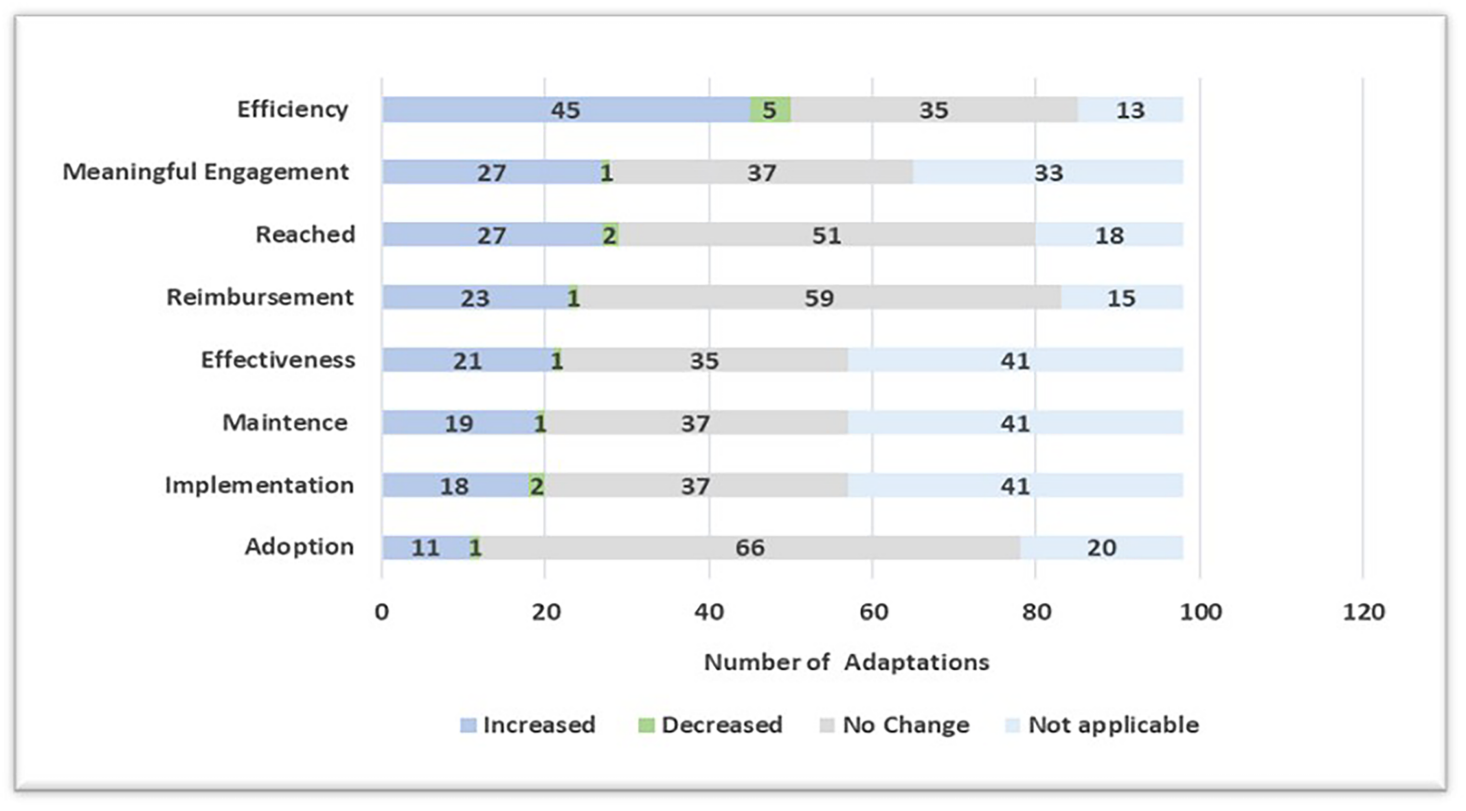
Perceived impact of adaptations on RE-AIM outcomes.

Figure 5 demonstrates the impact of the unique adaptations on using multiple RE-AIM implementation and effectiveness outcomes and rating them on an impact scale of increase, decrease, no change, or not applicable. In this paper, we assessed these impacts based on the following: reach (the number of patients engaged or reached), effectiveness (increases in the community testing at our location or other outcomes), adoption (the participation of clinic teams or staff in research activities), implementation (consistent delivery of quality of care or costs), maintenance (maintenance or sustainability of the patient within the intervention), reimbursement or financial implications, efficiency (getting more done faster or with less resources), and meaningful engagement. Most adaptations (*n* = 45, 45.9%) had a positive impact (i.e., increase) on the efficiency of the project delivery, meaningful engagement of partners, and reach of the type and number of community members through the program. Examples of these adaptations include CRC developed recruitment protocols to provide clear layout of processes for increased efficiency; tablets used by participants to fill out study survey must be connected to VPN at the start of the day in order to access multilingual tool (allows participants to access survey in Spanish) for an increase in meaningful engagement; and expansion of program eligibility to the larger San Ysidro region for increased reach. Negative impact was reported for a small number of adaptations (*n* = 14, 14.3%). The most common impact outcome where negative impact was identified was a decrease in efficiency. An example of an adaptation that resulted in reduced efficiency was relocating the recruitment site, shifting staff from inside the clinic courtyard to outside near the parking lot.

Adaptations can have multiple impacts. The adaptation that had the impact on the highest number of impact outcomes included changing testing hours from 9am to 3pm every Tuesday, Wednesday, and Thursday to expanding to five times a week Monday–Thursday 8:30am–3:00pm and Friday 9:00am–12:00pm. Expanding operation hours positively impacted reach, effectiveness, adoption, implementation, maintenance, efficiency, meaningful engagement, and reimbursement or financial implications for practice. Furthermore, there were 11 (11.2%) adaptations where no positive or negative change was detected. These adaptations included: the clinic hiring a new research assistant to help the team; the schedule for providing rapid antigen testing was modified to help team manage short-staffed days; and updating the project's website to include community events the team would support. Finally, there were adaptations (*n* = 2, 2.0%) that were determined to have only negative impact [i.e., decrease in one or multiple impact outcomes without any positive impact (increase) on other outcomes]. An example of an adaptation that only had negative impacts occurred during the project's implementation phase. Participants were asked to complete two separate COVID-19 tests as part of a pilot program with the University's partnering lab. This affected the workflow for the testing site by increasing testing wait times for participants and affecting the efficiency of testing site operations during the pilot program.

## Discussion

Using the RE-AIM expanded FRAME, we were able to systematically and prospectively capture adaptations, analyze, and determine the impact of these adaptations in our partner engaged CO-CREATE project (10, 24). This allowed for real time capturing and understanding of how our testing project iteratively changed to acknowledge and accommodate the realities of the COVID-19 pandemic and changing needs and priorities of the community. We documented a total of 98 unique adaptations with most of them identified during the implementation phase. Most adaptations were initiated by Clinical Research Coordinator and were focused on changing on-site testing operations based on Aim 2 key elements, many of which occurred during the implementation stage. These adaptations added to meaningful engagement and focused on the effectiveness of the project for all. Prior studies that used similar methods to document adaptations found that interactions between partners were effective in implementing and co-creating a successful study (6, 25, 26). Listening to community members, staff and partners enhanced the delivery of COVID-19 tests to a highly impacted community (27).

While for most of our variables we used the definitions proposed by Stirman and colleagues, we decided to use a different conceptualization of planned vs. unplanned adaptations. In our study, we used the terminology of planned adaptations for changes that were made after careful consideration of the implementation challenge. This was operationalized as “the adaptation being done after thoughtful discussion with at least one team member to address an issue.” We found in our prior work that this conceptualization of adaptations is more practical in this real world highly dynamic implementation study where the distinction between pre-implementaiton and implementation is less distinct. Using this definition, we found that most adaptations in our project were classified as planned adaptations. This is similar to many other studies where the definition of planned adaptations is broad and refers to any change where the team meaningfully discussed and decided on the change based on information/data (vs. made changes without discussion). If the definition of planned adaptation is limited to changes that occur pre-implementation (by design), our study would have classified most adaptations as unplanned.

Collecting data at different time points of the project allowed us to capture how our study was changing due to external factors associated with various COVID-19 variant waves. The Omicron variants brought many adaptations to on-site testing operations, as this milestone can be seen through various quarters of the project (Quarters 3–6) as seen in Figure 3. For example, the increase in COVID-19 cases during the Omicron Waves impacted how staff would return test results to participants and operational methods on-site due to high testing demands. Although the most frequent milestones were related to non-pandemic and non-milestone adaptations, the variability of adaptation milestones demonstrates the different factors impacting the project and the need to adapt.

Few studies to date have systematically documented the impact of adaptations on various implementation and effectiveness outcomes. Prior work, such as Wiltsey Stirman et al.'s FRAME framework (2013, 2019), has provided a structured approach to tracking and categorizing adaptations in evidence-based interventions, particularly in psychotherapies. While the expanded 2019 FRAME incorporates the intent behind modifications (e.g., improving feasibility, reducing costs), it does not offer a method to assess their actual impact on implementation or effectiveness outcomes. Similarly, Escoffery et al. (28) have contributed valuable descriptive research on the types and frequency of adaptations in public health interventions, but their work does not systematically evaluate the consequences of these modifications. These gaps highlight the need for approaches that go beyond documenting and classifying adaptations to systematically assessing their effects on key implementation and effectiveness outcomes. A key innovation in this study was the use of a team-based consensus approach to assess the impact of adaptations on a wide range of RE-AIM outcomes. While this method does not rely on systematically collected quantitative data for each outcome, it leverages the collective experience of the implementation and research team to gauge adaptation impact, incorporating study data where available (e.g., changes in recruitment, engagement, and completion of recommended actions). This approach builds on prior adaptation frameworks by explicitly linking adaptations to observed implementation outcomes. However, our findings also revealed that some adaptations did not fit neatly within the predefined impact categories, underscoring the need for further refinement of adaptation-outcome alignment. Future work will refine these classifications, explicitly aligning the intended purpose of adaptations with their actual impact—an approach that can help address ongoing challenges in adaptation research and move the field beyond descriptive documentation.

This study has limitations. While our team followed a longitudinal, prospective approach with team-based validation to documenting adaptations, we might have missed some adaptations due to the fast pace and competing data collection demands of this study (29). The nature of the CO-CREATE project did not allow for a controlled environment to systematically evaluate the impact of adaptations, which is still a new and unresolved area of the field (3). Furthermore, while we included a number of equity-relevant component into our documentation forms, more comprehensive assessment of the impact of adaptations on equity-relevant outcomes and resources and cost would be especially important in future work. Specifically, the extended RE-AIM reporting criteria and conceptualization could serve as a basis for this expansion (18, 24). In addition, the multi-method approach used is comprehensive, theoretically driven, and actionable. However, it required significant resources from study staff and partners who, while perceiving it as a beneficial endeavor, required investment of time and study resources not explicitly earmarked in the project proposal. As noted earlier, the documentation of impact of adaptations is novel and will benefit from further refinement. Finally, we need to further increase the efficiency of our documentation process, including refining the specification on what changes should and should not be documented.

## Conclusion

We were able to systematically and prospectively document and analyze adaptations over a two-year period and reflect on the impact of these modifications. The use of multiple sources to collect this longitudinal data allowed us to validate and obtain information to see in real time how a project was able to adapt to both internal and external factors related to a pandemic. Future studies need to expand on capturing the impact of adaptations both at a short-term and long-term period.

## Data Availability

The raw data supporting the conclusions of this article will be made available by the authors, without undue reservation.
