# Supplementary material for: Understanding adaptations in a community-vetted COVID-19 testing program
Source: Front Health Serv. 2025 Apr 7;5:1408940. doi: 10.3389/frhs.2025.1408940 (PMC12009949; doi:10.3389/frhs.2025.1408940)
Supplement: Supplementary file 1 [file Table1.pdf]

Supplementary Table 1

Tool Field Name	Notes
Who is creating this Adaptation?	
1. Analyst/ Reporter<br>○ UCSD CRC(s)<br>○ UCSD Investigator(s)<br>○ CSAB<br>○ SYH Research Staff<br>○ SYH Investigator(s)<br>○ SYH Provider(s)<br>○ Other (Please Specify)	Who is reporting adaptation in REDCap<br>Specify other analyst/ reporter
2. Identifier<br>• UCSD Research Staff<br>• UCSD Investigator(s)<br>• CSAB<br>• SYH Research Staff<br>• SYH Investigator(s)<br>• SYH Provider(s)<br>• Other (Please Specify)	Who identified adaptation, may be same as Analyst/ Reporter<br>Specify other identifier
3. Site Code<br>• MCHC<br>• SYHC<br>• Community Sites<br>• Across Project<br>• CSAB<br>• Other (Please Specify)	Specify other site
4. Date Recorded	Date when record was made
5. Date of Change	(MM/YYYY)<br>When change was implemented
Adaptation Information	
6. Adaptation Title	Ex: change in recruitment
7. Adaptation Brief summary	Summary of what was changed<br><br>Brief description of the adaptation that was made (try to keep it to 1-2 sentences but provide enough context that it stands alone. For example: recruitment criteria was changed to include all patients with XX code as well.
8. Was it planned or unplanned?<br><br>• Planned<br>• Unplanned	Planned - discussed with team & made decision based on data/experience<br><br>Unplanned - change was made without shared discussion and agreement and possibly without looking at data
Details About Adaptation	

9. What element was changed?<br>• The setting<br>• The format<br>• Personnel involved<br>• How the intervention/ program is presented/ delivered- how core components are operationalized<br>• Other (Please Specify)	Select all that apply<br>Format: example: in person changed to tele<br>Specify other type of element changed
10. What was the type of change?	Select all that apply<br>Specify other type of change
AIM 1 & AIM 2: Research and Logistical Process	
11. Which core component is this change related to?<br>• AIM 1: Partner Engagement and Community Needs/Assets Assessment<br>• AIM 2: COVID-19 Testing<br>• AIM 1 & AIM 2: Research and Logistical Process<br>• Other (Please Specify)	Please specify OTHER core component that change is related to?
12. AIM 1: Partner Engagement & Community Needs/Assets Assessment Subcategory<br>• CSAB<br>• SYH AIM 1 Data Activities<br>• Data Analysis for AIM 1<br>• Other (Please Specify)	Please specify OTHER AIM 1: Partner Engagement & Community Needs/Assets Assessment<br><br>Community & Scientific Advisory Board (CSAB):<br>Identification & Recruitment of members<br>Documentation of CSAB<br>CSAB meeting - theory of change, appreciative inquiry CSAB meeting - members, processes<br><br>AIM 1 Data Activities:<br>SYH recruitment and data collection for Aim 1 survey<br>SYH recruitment and data collection for brainwriting Social media/website marketing/recruitment<br><br>AIM 2: COVID-19 Testing:<br>SYH & Community Testing (testing processes, recruitment for testing)<br>Data Collection for AIM 2 (surveys)<br>Data Analysis for AIM 2
13. AIM 1 & AIM 2: Research and Logistical Process<br>• IRB<br>• Personnel<br>• Incentives (e.g., changed from none to weekly raffle- For Aim 2;	

Use of Amazon cards temporarily for AIM 1)**<br>• Translations	
14. Please specify type of Translations<br>• English written<br>• English live<br>• Spanish written<br>• Spanish live	
Who Is Involved in This Adaptation Change?	
15. Who was responsible for initiating this change?<br>• Entire or most of team<br>• CRC<br>• Other Researcher<br>• CSAB<br>• Principle or Co-Principal Investigator(s)<br>• SYH Research Staff<br>• SYH Investigator(s)<br>• SYH Provider(s)<br>• SYH Study Participant<br>• SYH Non-Study Participant<br>• SYH Administration/ Organization leaders<br>• SYH Residents/ Community Members<br>• Other (Please Specify)	Please specify OTHER that was responsible for initiating change
When and Why Was This Adaptation Made?	
16. When during CO-CREATE program was this adaptation made?<br>○ Pre Implementation (AIM 1)<br>○ Early Implementation (AIM 2)<br>○ Implementation (AIM 2)<br>○ Late Implementation (AIM 2)	
17. Which stage of the COVID-19 pandemic is this adaptation associated with?<br>• Before testing started<br>• Delta 2 (July - Oct 2021)<br>• Omicron (Dec 2021 - Mar 2022)<br>• Omicron 2 (June - Aug 2022)<br>• At home rapid test availability through SYH (Feb 17, 2022)<br>• CDPH rapid test availability on site (July 5, 2022)<br>• Free testing kits available - USPS (Jan 2022)<br>• Testing guideline changes, accepting rapid test results (schools)	Please specify OTHER stage of the COVID-19 pandemic this adaptation is associated with

• Testing guideline changes, accepting rapid test results (work) Testing guideline changes, border opening (travel) (Nov 2021)<br>• Testing guideline changes, accepting rapid test results (travel)<br>• Recommendations around preventive behaviors<br>• Vaccine/booster availability (multiple dates)<br>• Weather changes (multiple dates)<br>• Staffing availability (multiple dates)<br>• Other (Please Specify)<br>• None of the above	
18. How or on what basis was this change made?<br>• Based on our vision or values<br>• Based on our framework (for example PCMH) Based on our knowledge or experience of working with patients<br>• Based on QI data, summary information or results<br>• Based on pragmatic/practical considerations (ex: is this the only way it could work?)<br>• Based on financial incentives/payment<br>• Based on feedback or suggestions<br>• Other (Please Specify)	Please specify OTHER basis why change was made
19. Why was this change made?<br>• To increase the number or type of patients contacted (reach)<br>• To enhance the impact or success of the intervention for all or important subgroups (effectiveness)<br>• To make it possible to involve more teams, team members or staff (adoption)<br>• To deliver intervention more consistently; better for practice, patient flow or EHR (implementation)<br>• For practical reasons (implementation)	reason/what prompted need for adaptation<br><br>Please specify OTHER reason why change was made

• To institutionalize or sustain the intervention (maintenance)<br>• To respond to external pressures or policy<br>• To save money or other resources (implementation)<br>• Other (Please Specify)	
20. Was this adaptation a result of EXTERNAL FACTORS or INTERNAL ISSUES?<br>○ External<br>○ Internal	External factors - related to non-research team/processes<br><br>Internal issues - related to research team/processes
What Was the Short Term Impact of This Change?	
21. Number or type of patients engaged reached<br>○ Increased<br>○ Decreased<br>○ No Change<br>○ Not Applicable	Aim 1: Reach- increased/decreased survey/interview completion by patients/community members
22. Increase community testing or other outcome<br>○ Increased<br>○ Decreased<br>○ No Change<br>○ Not Applicable	Effectiveness
23. Participation of teams or staff<br>○ Increased<br>○ Decreased<br>○ No Change<br>○ Not Applicable	Aim 1: Adoption- increased/decreased survey/ interview completion by SYH staff/providers
24. Consistent delivery of quality of care or costs<br>○ Increased<br>○ Decreased<br>○ No Change<br>○ Not Applicable	Implementation- engagement can apply to Aim1: CSAB| Aim2: Testing, privacy etc.
25. Maintenance or sustainability of the patient within the intervention<br>○ Increased<br>○ Decreased<br>○ No Change<br>○ Not Applicable	Maintenace
26. Reimbursement or financial implications for the practice<br>○ Increased<br>○ Decreased<br>○ No Change<br>○ Not Applicable	Aim 1: Reimbursement and financial implications - impact on study finances

27. Efficiency<br>o Increased<br>o Decreased<br>o No Change<br>o Not Applicable	Aim 1: More surveys/ interviews completed with less effort| Aim 2: getting more done faster or with less resources
28. Meaningful engagement<br>o Increased<br>o Decreased<br>o No Change<br>o Not Applicable	The quality and/or documentation of engagement
Was this Adaptation Successful?	
29. Was the adaptation successfully implemented?<br><br>o Yes<br>o No	If YES, please explain why<br><br>If NO, please explain why
30. Please include any other important details	
31. Was it reviewed by the Adaptations Team?<br>o Yes<br>o No	
