# Supplementary material for: Understanding adaptations in a community-vetted COVID-19 testing program
Source: Front Health Serv. 2025 Apr 7;5:1408940. doi: 10.3389/frhs.2025.1408940 (PMC12009949; doi:10.3389/frhs.2025.1408940)
Supplement: Supplementary file 2 [file Table2.pdf]

Supplementary Table 2: Detailed list and characteristics of adaptation documented in the CO-CREATE study

**Adaptation Title**	**Adaptation Brief Summary**	**Was it planned or unplanned?**	**What element was changed?**	**What was the type of change?**	**Which core component is this change related to?**	**Who was responsible for initiating this change?**	**When during CO-CREATE program was this adaptation made?**	**How or on what basis was this change made?**	**Why was this change made?**	**What Was the Impact of This Change?**
Adaptation name for reference	Brief description of the adaptation	Planned<br>Unplanned	The setting<br>The format<br>Personnel involved<br>Target population<br>How the intervention/program is presented/delivered- how core components are operationalized<br>Other	Tailoring to individuals<br>Adding a component<br>Removing a component<br>Condensing a component<br>Extending a component<br>Substituting for a component<br>Changing the order of components<br>Repeating a component<br>Integrating with other programs we are doing<br>Loosening the structure or protocol<br>Otherwise changing the intervention<br>Other	AIM 1: Partner Engagement and Community Needs/Assets Assessment<br><br>*Community & Scientific Advisory Board (CSAB)*<br><br>*San Ysidro Health (SYH) AIM 1 Data Activities*<br><br>*Data Analysis for AIM 1*<br><br>*Other*<br>AIM 2: COVID-19 Testing<br>AIM 1 & AIM 2: Research and Logistical Process<br><br>*Institutional Review Board (IRB)*<br><br>*Personnel*<br><br>*Incentives*<br><br>*Translations*<br>*English written*<br>*English live*<br>*Spanish written*<br>*Spanish live*<br>Other	Entire or most of team<br>Clinical Research Coordinator (CRC)<br>Researcher<br>Community & Scientific Advisory Board (CSAB)<br>Principle or Co-Principle Investigator(s)<br>San Ysidro Health (SYH) Research Staff<br>San Ysidro Health Investigators<br>San Ysidro Health Provider(s)<br>San Ysidro Health Study Participant<br>San Ysidro Health Non-Study Participant<br>San Ysidro Health Administration/ Organization<br>San Ysidro Residents/ Community Members<br>Other	Pre-Implementation (AIM 1)<br>Early Implementation (AIM 2)<br>Implementation (AIM 2)<br>Late Implementation (AIM 2)	Based on our values<br>Based on our framework<br>Based on our knowledge or experience of working with patients<br>Based on QI data, summary information or results<br>Based on pragmatic/ practical considerations<br>Based on financial incentives/ payments<br>Based on feedback or suggestions<br>Other	To increase the number or type of patients contacted (reach)<br>To enhance the impact or success of the intervention for all or important subgroups (effectiveness)<br>To make it possible to involve more teams, team members or staff (adoption)<br>To deliver intervention more consistently; better for practice, patients flow or HER (implementation)<br>For practical reasons (implementation)<br>To institutionalize the intervention (maintenance)<br>To respond to external pressures or policy<br>To save money or other resources (implementation)<br>Other	Number or type of patients engaged reached<br><br>*Increase*<br>*Decrease*<br>*No change*<br>Increase community testing or other outcome (effectiveness)<br><br>*Increase*<br>*Decrease*<br>*No change*<br>Participation of teams or staff<br><br>*Increase*<br>*Decrease*<br>*No change*<br>Consistent delivery of quality of care or costs<br><br>*Increase*<br>*Decrease*<br>*No change*<br>Maintenance or sustainability of the patient within the intervention (maintenance)<br><br>*Increase*<br>*Decrease*<br>*No change*<br><br>Reimbursement or financial implications for the practice<br><br>*Increase*<br>*Decrease*

										No change<br>Efficiency<br><br>*Increase*<br>*Decrease*<br>*No change*<br>Meaningful engagement<br><br>*Increase*<br>*Decrease*<br>*No change*

Changed recruitment location	Added recruitment locations to 2nd floor exits and oppose side of Maternal and Child Health Clinic (MCHC) entrance line due to target population exiting from different side of building	Planned	The setting<br><br>How the intervention/ program is presented/ delivered - how core components are operationalized	Tailoring to individuals<br><br>Extending a component	AIM 1: Partner Engagement and Community Needs/Assets Assessment<br><br>*San Ysidro Health (SYH) AIM 1 Data Activities*	Entire or most of team	Pre-implementation (AIM 1)	Based on our knowledge or experience of working with patients<br><br>Other: Based on observations	To increase the number or type of patients contacted (reach)<br><br>To enhance the impact or success of the intervention for all or important subgroups (effectiveness)	Number or type of patients engaged reached - Increased<br><br>Participation of teams or staff - No change<br><br>Reimbursement or financial implications for the practice - No change<br><br>Efficiency - Increased
Recruitment Outreach	Included mass email/text blast to increase recruitment response.	Planned	How the intervention/ program is presented/ delivered - how core components are operationalized	Adding a component<br><br>Extending a component	AIM 1: Partner Engagement and Community Needs/Assets Assessment<br>*SYH AIM 1 Data Activities*	Entire or most of team	Pre-implementation (AIM 1)	Based on our knowledge or experience of working with patients<br><br>Based on feedback or suggestions<br><br>Other: Based on observations	To increase the number or type of patients contacted (reach)<br><br>To enhance the impact or success of the intervention for all or important subgroups (effectiveness)	Number or type of patients engaged reached- Increased<br><br>Participation of teams or staff- No change<br><br>Reimbursement or financial implications for the practice - No change<br><br>Efficiency - Increased

Changed recruitment location	Moved from inside SYH courtyard to outside SYH by entrance/parking lot	Planned	The setting	Condensing a component	AIM 1: Partner Engagement and Community Needs/Assets Assessment *SYH AIM 1 Data Activities*	SYH Administration/ Organization leaders	Pre-implementation (AIM 1)	Based on feedback or suggestions<br><br>Other: SYH Admin conducting vaccinations in courtyard and cannot accommodate UCSD staff and patients in same area	To respond to external pressures or policy	Number or type of patients engaged reached- Decreased<br><br>Participation of teams or staff- No change<br><br>Reimbursement or financial implications for the practice - No change<br><br>Efficiency - Decreased
Gift Cards	Human compensation gift cards was changed from Walmart/Target/ Amazon gift cards to UCSD Vanilla visa gift cards. Change was made b/c UCSD cards have lower fees and participants have more flexibility on where it can be used	Planned	Other: process refined due to participant feedback as well as CRC (AME) experience with UCSD human compensation	Substituting a component	AIM 1: Partner Engagement and Community Needs/Assets Assessment *SYH AIM 1 Data Activities*	CRC	Pre-implementation (AIM 1)	Based on financial incentives/ payment<br><br>Based on feedback or suggestions<br><br>Other: CSAB recommendation	To enhance the impact or success of the intervention for all or important subgroups (effectiveness)<br><br>To save money or other resources (implementation)	Number or type of patients engaged reached- No change<br><br>Participation of teams or staff- No change<br><br>Reimbursement or financial implications for the practice – Increased<br><br>Efficiency – No change
Gift Cards Order Challenge	Financial system change and request was met with multiple issues regarding funding#/approval flow and delayed compensation to participants	Unplanned	How the intervention/ program is presented/ delivered - how core components are operationalized	Substituting a component	AIM 1: Partner Engagement and Community Needs/Assets Assessment *SYH AIM 1 Data Activities*	CRC	Pre-implementation (AIM 1)	Based on financial incentives/ payment	To save money or other resources (implementation)	Number or type of patients engaged reached- No change<br><br>Participation of teams or staff- No change<br><br>Reimbursement or financial implications for the practice – Increased<br><br>Efficiency – Increased

Recruitment Material	Recruitment flyers were revised to be more clear and concise QR codes were added for interested participants to easily access the survey	Planned	How the intervention/ program is presented/ delivered - how core components are operationalized	Tailoring to individuals<br><br>Condensing a component	AIM 1: Partner Engagement and Community Needs/Assets Assessment *SYH AIM 1 Data Activities*	CRC	Pre-implementation (AIM 1)	Based on our knowledge or experience of working with patients<br><br>Based on feedback or suggestions	To increase the number or type of patients contacted (reach)<br><br>To enhance the impact or success of the intervention for all or important subgroups (effectiveness)	Number or type of patients engaged reached- Increased<br><br>Participation of teams or staff- No change<br><br>Reimbursement or financial implications for the practice – No change<br><br>Efficiency – Increased
Development of Recruitment Protocols	CRC developed recruitment protocols to provide clear layout of processes for various tasks including gift card ordering/tracking/distribution; participant survey delivery methods; recruitment methods; REDCap instructions with more being developed	Planned	How the intervention/ program is presented/ delivered - how core components are operationalized	Extending a component	AIM 1: Partner Engagement and Community Needs/Assets Assessment *SYH AIM 1 Data Activities*	CRC	Pre-implementation (AIM 1)	Based on our vision or values<br><br>Based on pragmatic/practical considerations (i.e., is this the only way it could work)	To increase the number or type of patients contacted (reach)<br><br>To enhance the impact or success of the intervention for all or important subgroups (effectiveness)<br><br>To deliver intervention more consistently; better for practice, patient flow or EHR (implementation)	Number or type of patients engaged reached- No change<br><br>Participation of teams or staff- No change<br><br>Reimbursement or financial implications for the practice – No change<br><br>Efficiency – Increased
FAQ Sheet	FAQ sheet developed to help answer any questions. Posted on website and used during patient/provider interviews.	Planned	How the intervention/ program is presented/ delivered - how core components are operationalized	Adding a component	AIM 1: Partner Engagement and Community Needs/Assets Assessment *SYH AIM 1 Data Activities*	CRC	Pre-implementation (AIM 1)	Based on feedback or suggestions<br><br>Other: Based on provider pilot interview feedback	To enhance the impact or success of the intervention for all or important subgroups (effectiveness)<br><br>To deliver intervention more	Number or type of patients engaged reached- No change<br><br>Participation of teams or staff- No change

									consistently; better for practice, patient flow or EHR (implementation)	Reimbursement or financial implications for the practice – No change<br><br>Efficiency – Increased
Inclusion of Free Response in Survey	CSAB meeting surveys were revised to include a free response section to allow participants to provide valuable feedback and suggestions	Planned	How the intervention/ program is presented/ delivered - how core components are operationalized	Extending a component	AIM 1: Partner Engagement and Community Needs/Assets Assessment *CSAB*	CSAB	Pre-implementation (AIM 1)	Based on feedback or suggestions<br><br>Other: CSAB recommendation	To enhance the impact or success of the intervention for all or important subgroups (effectiveness)	Number or type of patients engaged reached- No change<br><br>Participation of teams or staff- No change<br><br>Reimbursement or financial implications for the practice – No change<br><br>Efficiency – Increased
Expansion of Program Eligibility to SY Community	Expansion of program eligibility to the larger San Ysidro region. CSAB recommendation to increase testing to not just SYH patients, but everyone in the community.	Planned	The target population<br><br>How the intervention/ program is presented/ delivered - how core components are operationalized	Tailoring to individuals<br><br>Extending a component	AIM 2: COVID-19 Testing	CSAB	Early Implementation (AIM 2)	Based on our knowledge or experience of working with patients<br><br>Based on feedback or suggestions<br><br>Other: CSAB recommnedation	To increase the number or type of patients contacted (reach)<br><br>To enhance the impact or success of the intervention for all or important subgroups (effectiveness)	Number or type of patients engaged reached- Increased<br><br>Effectiveness- Increased<br><br>Participation of teams or staff- Increased<br><br>Implementation - No change<br><br>Maintenance- Increased<br><br>Reimbursement or financial implications for the practice – No change<br><br>Efficiency – Increased
Return of Results	Due to SYH protocols, Dr. Laurent and Caroline do not have ability to	Planned	How the intervention/ program is presented/	Removing a component	AIM 2: COVID-19 Testing	SYH Administration/ Organization leaders	Early Implementation (AIM 2)	Based on pragmatic/ practical considerations (ex: is this the	To respond to external pressures or policy	Number or type of patients engaged reached- Increased

	schedule an appointment for patients requesting follow-up appt with a SYH provider. Patients will have to call direct line to call center to schedule an appointment.		delivered - how core components are operationalized					only way it could work?		Participation of teams or staff- Increased<br><br>Reimbursement or financial implications for the practice – No change<br><br>Efficiency – Increased
Social Media Adaptation	Tagging relevant user/institutions to Instagram/ Twitter posts to improve outreach. Additional tags were added to posts	Planned	How the intervention/ program is presented/ delivered - how core components are operationalized	Tailoring to individuals<br><br>Extending a component	AIM 2: COVID-19 Testing	Other: Intern	Early Implementation (AIM 2)	Based on our knowledge or experience of working with patients<br><br>Based on QI data, summary information or results<br><br>Based on pragmatic/ practical considerations (i.e., is this the only way it could work?)	To increase the number or type of patients contacted (reach)<br><br>To enhance the impact or success of the intervention for all or important subgroups (effectiveness)	N/A
Social Media Adaptation	Linking website to each post	Planned	How the intervention/ program is presented/ delivered - how core components are operationalized	Extending a component	AIM 2: COVID-19 Testing	Other: Intern	Early Implementation (AIM 2)	Based on feedback or suggestions	To increase the number or type of patients contacted (reach)<br><br>To enhance the impact or success of the intervention for all or important subgroups (effectiveness)<br><br>To deliver intervention more consistently; better for practice, patient flow or EHR (implementation)	N/A

									For practical reasons (implementation)	
Social Media Adaptation	Following users on social media platforms expecting users would follow back	Planned	The target population	Extending a component	AIM 2: COVID-19 Testing	Other: Intern	Early Implementation (AIM 2)	Based on our knowledge or experience of working with patients<br><br>Based on pragmatic/ practical considerations (i.e., is this the only way it could work?)<br><br>Based on feedback or suggestions	To increase the number or type of patients contacted (reach)<br><br>To enhance the impact or success of the intervention for all or important subgroups (effectiveness)	Number or type of patients engaged reached- no change
Clarification of participant status	REDCap surveys were updated to provide clarification between a prenatal patient or a pediatric caregiver. Prenatal patient categorized as 3 months postpartum.	Planned	How the intervention/ program is presented/ delivered - how core components are operationalized	Tailoring to individuals<br><br>Adding a component	AIM 1: Partner Engagement and Community Needs/Assets Assessment *SYH AIM 1 Data Activities*	CRC	Pre-implementation (AIM 1)	Based on our knowledge or experience of working with patients<br><br>Based on pragmatic/ practical considerations (i.e., is this the only way it could work?)<br><br>Based on feedback or suggestions	To enhance the impact or success of the intervention for all or important subgroups (effectiveness)<br><br>To deliver intervention more consistently; better for practice, patient flow or EHR (implementation)<br><br>For practical reasons (implementation)	Number or type of patients engaged reached- No change<br><br>Participation of teams or staff- No change<br><br>Reimbursement or financial implications for the practice – No change<br><br>Efficiency – Increased
Registration Workflow	Move from REDCap during registration to using OneDrive Excel doc and completing REDCap data transfer after results are logged on sheet	Planned	The format	Substituting for a component<br><br>Changing the order of components	AIM 2: COVID-19 Testing	CRC	Early Implementation (AIM 2)	Based on our knowledge or experience of working with patients<br><br>Based on pragmatic/ practical considerations (i.e., is this the only way it could work?)	To enhance the impact or success of the intervention for all or important subgroups (effectiveness)<br><br>For practical reasons (implementation)	Number or type of patients engaged reached- No change<br><br>Effectiveness- No change<br><br>Participation of teams or staff- No change<br><br>Implementation - No change

										Maintenance- No change<br><br>Reimbursement or financial implications for the practice – No change<br><br>Efficiency – Increased<br><br>Meaningful engagement- No change
REDCap Language Module	Implementation of REDCap language module to easily select English or Spanish survey for participants and for easier data analysis because variable fields are now captured in one export	Planned	The format<br><br>How the intervention/ program is presented/ delivered - how core components are operationalized	Adding a component<br><br>Extending a component	AIM 2: COVID-19 Testing	CRC	Early Implementation (AIM 2)	Based on pragmatic/ practical considerations (i.e., is this the only way it could work?	To increase the number or type of patients contacted (reach)<br><br>To deliver intervention more consistently; better for practice, patient flow or EHR (implementation)<br><br>For practical reasons (implementation)<br><br>Other: to analyze data more easily with less back end data cleaning and merging of documents	Number or type of patients engaged reached- No change<br><br>Effectiveness- No change<br><br>Participation of teams or staff- No change<br><br>Implementation - No change<br><br>Maintenance- No change<br><br>Reimbursement or financial implications for the practice – No change<br><br>Efficiency – Increased<br><br>Meaningful engagement- No change
Inclusion of Vaccination Hesitancy Questions	Vaccine hesitancy questions included into survey to gain better knowledge of community experiences and hesitations towards vaccines,	Planned	How the intervention/ program is presented/ delivered - how core components are operationalized	Adding a component	AIM 2: COVID-19 Testing	Entire of most of team	Early Implementation (AIM 2)	Based on QI data, summary information or results<br><br>Based on feedback or suggestions	To enhance the impact or success of the intervention for all or important subgroups (effectiveness)<br><br>To deliver intervention more consistently;	Number or type of patients engaged reached- No change<br><br>Effectiveness- No change<br><br>Participation of teams or staff- No change

	specifically COVID-19								better for practice, patient flow or EHR (implementation)<br><br>For practical reasons (implementation)<br><br>To respond to external pressures or policy	Implementation - No change<br><br>Maintenance- No change<br><br>Reimbursement or financial implications for the practice – No change<br><br>Efficiency – No change<br><br>Meaningful engagement- No change
Re-ordering of survey questions	Survey questions are reorganized to improve flow of survey and to prioritize questions in order to reduce survey fatigue	Planned	The format<br><br>How the intervention/ program is presented/ delivered - how core components are operationalized	Changing the order of components	AIM 2: COVID-19 Testing	CRC<br><br>Principle or Co-Principle Investigator(s)	Early Implementation (AIM 2)	Based on our knowledge or experience of working with patients<br><br>Based on feedback or suggestions	To enhance the impact or success of the intervention for all or important subgroups (effectiveness)<br><br>To deliver intervention more consistently; better for practice, patient flow or EHR (implementation)<br><br>For practical reasons (implementation)	N/A
Improving Consenting and Interview Flow for Patient/Provider Interviews	Consenting process and interview questions condensed to increase efficiency of patient and provider interviews. This also allows more time for patient/provider feedback during interviews.	Planned	The format<br><br>How the intervention/ program is presented /delivered - how core components are operationalized	Condensing a component	AIM 1: Partner Engagement and Community Needs/Assets Assessment *SYH AIM 1 Data Activities Data Analysis for AIM 1*	CRC<br><br>Principle or Co-Principle Investigator(s)	Pre-implementation (AIM 1)	Based on our knowledge or experience of working with patients<br><br>Based on feedback or suggestions	To enhance the impact or success of the intervention for all or important subgroups (effectiveness)<br><br>To deliver intervention more consistently; better for practice, patient flow or EHR (implementation)	Number or type of patients engaged reached- No change<br><br>Effectiveness- No change<br><br>Participation of teams or staff- No change<br><br>Implementation - No change<br><br>Maintenance- No change

										Reimbursement or financial implications for the practice – No change<br><br>Efficiency – Increased<br><br>Meaningful engagement- Increased
Community Outreach	Exploring different areas within the San Ysidro community to expand access to testing and have option to test at different locations.	Planned	The target population	Extending a component	AIM 2: COVID-19 Testing	CRC	Early Implementation (AIM 2)	Based on our knowledge or experience of working with patients<br><br>Based on feedback or suggestions	To increase the number or type of patients contacted (reach)<br><br>To enhance the impact or success of the intervention for all or important subgroups (effectiveness)<br>To deliver intervention more consistently; better for practice, patient flow or EHR (implementation)<br><br>To institutionalize or sustain the intervention (maintenance)<br><br>To respond to external pressures or policy	N/A
Mobile Hot Spot	A mobile hot spot was identified as a need in order to access secure internet access outside MCHC and within community	Planned	Other: Tools used to ensure consistent access to applications needed for documentation/ registration of participants	Adding a component	AIM 2: COVID-19 Testing	CRC	Early Implementation (AIM 2)	Based on pragmatic/ practical considerations (i.e., is this the only way it could work?	To deliver intervention more consistently; better for practice, patient flow or EHR (implementation)<br><br>For practical reasons (implementation)	Efficiency- Increased

Layout of patient flow	Tables were rearranged to improve patient flow	Planned	The setting	Changing the order of components	AIM 2: COVID-19 Testing	CRC	Early Implementation (AIM 2)	Based on our knowledge or experience of working with patients<br><br>Based on feedback or suggestions	For practical reasons (implementation)	Efficiency-Increased
Streamline RoR Method of Contact	RoR method of contact was changed from preferred option to email as default method unless otherwise specified by participant	Planned	How the intervention/ program is presented/ delivered - how core components are operationalized	Condensing a component	AIM 2: COVID-19 Testing	CRC	Early Implementation (AIM 2)	Based on pragmatic/ practical considerations (i.e., is this the only way it could work?<br><br>Based on our knowledge or experience of working with patients	To enhance the impact or success of the intervention for all or important subgroups (effectiveness)<br><br>To deliver intervention more consistently; better for practice, patient flow or EHR (implementation)<br><br>For practical reasons (implementation)	N/A
All CRC's to have access to NextGen	To improve patient flow and distribution of task, UCSD team members will also have access to NextGen	Planned	Personnel involved	Extending a component	AIM 2: COVID-19 Testing	CRC<br><br>SYH Research staff	Early Implementation (AIM 2)	Based on pragmatic/ practical considerations (i.e., is this the only way it could work?<br><br>Based on feedback or suggestions	To increase the number or type of patients contacted (reach)<br><br>To enhance the impact or success of the intervention for all or important subgroups (effectiveness)<br><br>To make it possible to involve more teams, team members or staff (adoption)<br><br>To deliver intervention more consistently; better for practice, patient	Efficiency-Increased

									flow or EHR (implementation)<br><br>For practical reasons (implementation)	
Requirement of additional documentation for patient registration	Additional forms to be completed by non SYH participants in order to be registered as a SYH patient to receive a test	Planned	How the intervention/program is presented/delivered - how core components are operationalized	Adding a component	AIM 2: COVID-19 Testing	SYH Research staff<br><br>SYH Administration/ Organization leaders	Early Implementation (AIM 2)	Based on our knowledge or experience of working with patients<br><br>Based on feedback or suggestions	To respond to external pressures or policy	Efficiency- Decreased
Change in Testing Goals	Daily testing goals are decreased due to county wide testing statistics and what is a feasible daily/weekly/monthly goal based on staff and resources available	Planned	The target population<br><br>Other: Number of enrolled participants per day to decrease	Condensing a component	AIM 2: COVID-19 Testing	CRC	Early Implementation (AIM 2)	Based on QI data, summary information or results	For practical reasons (implementation)	Efficiency- No change
Inclusion of Raffle Option	The inclusion of a raffle as an incentive was added into the research plan and consent forms to encourage participation in survey	Planned	How the intervention/ program is presented/ delivered - how core components are operationalized	Adding a component	AIM 1 & AIM 2: Research and Logistical Process<br>*IRB*<br>*Incentives*	Principle or Co-Principle Investigator(s)	Early Implementation (AIM 2)	Based on our knowledge or experience of working with patients<br><br>Based on financial incentives/ payment	To increase the number or type of patients contacted (reach)<br><br>To enhance the impact or success of the intervention for all or important subgroups (effectiveness)	Efficiency- No change
Raffle Incentive Changed to Gift Cards per survey	The raffle incentive was replaced with gift card incentives ($20 - 1st survey; $10 - all repeat surveys) to improve participation in surveys and return visits	Planned	How the intervention/ program is presented/ delivered - how core components are operationalized	Substituting a component	AIM 1 & AIM 2: Research and Logistical Process<br>*Incentives*	CRC	Early Implementation (AIM 2)	Based on financial incentives/ payment	To increase the number or type of patients contacted (reach)<br><br>To enhance the impact or success of the intervention for all or important subgroups (effectiveness)	Number or type of patients engaged reached- Increased<br><br>Effectiveness- Increased<br><br>Participation of teams or staff- Increased<br><br>Implementation - No change

										Maintenance- Increased<br><br>Reimbursement or financial implications for the practice – Increased<br><br>Efficiency – No change
Option for In-Person Patient Interviews	Patients provided option of in-person interviews if unreliable access to internet/ computer/zoom application	Planned	The setting	Tailoring to individuals<br><br>Adding a component	AIM 1: Partner Engagement and Community Needs/Assets Assessment *SYH AIM 1 Data Activities*	CRC	Pre-implementation (AIM 1)	Based on our knowledge or experience of working with patients<br><br>Based on feedback or suggestions	To increase the number or type of patients contacted (reach)<br><br>To enhance the impact or success of the intervention for all or important subgroups (effectiveness)	Number or type of patients engaged reached- Increased<br><br>Participation of teams or staff- No change<br><br>Reimbursement or financial implications for the practice – No change<br><br>Efficiency- Decreased
Change of workflow with edits to patient/prov ide flow scripts and videos	Video recordings of patient and provider flows will no longer be included in patient/provider interviews. Change was made in order reduce admin time in edits of video/recordings	Planned	The format<br><br>How the intervention/ program is presented/ delivered - how core components are operationalized	Removing a component	AIM 1: Partner Engagement and Community Needs/Assets Assessment *SYH AIM 1 Data Activities*	CRC<br><br>Principle or Co-Principle Investigator(s)<br><br>SYH Research staff	Pre-implementation (AIM 1)	Based on pragmatic/ practical considerations (i.e., is this the only way it could work?<br><br>Based on feedback or suggestions	For practical reasons (implementation)	Number or type of patients engaged reached- No change<br><br>Participation of teams or staff- No change<br><br>Reimbursement or financial implications for the practice – No change<br><br>Efficiency-No change
Increase length of CSAB meetings	Increase length of meeting from 1.5 to 2 hrs	Planned	The format	Extending a component	AIM 1: Partner Engagement and Community Needs/Assets Assessment *CSAB*	CSAB	Pre-implementation (AIM 1)	Based on feedback or suggestions	To enhance the impact or success of the intervention for all or important subgroups (effectiveness)	Number or type of patients engaged reached- No change

									To make it possible to involve more teams, team members or staff (adoption)	Participation of teams or staff- No change<br><br>Reimbursement or financial implications for the practice – Increased<br><br>Efficiency-No change<br><br>Meaningful engagement- Increased
Changes in interpretation/translation done during CSAB meetings	Changes to process of interpretation and translation during meetings was changed to allow translator/interpreter more time to listen, respond, and given breaks to catch up on materials during meeting	Planned	The format<br><br>How the intervention/ program is presented/ delivered - how core components are operationalized	Tailoring to individuals	AIM 1: Partner Engagement and Community Needs/Assets Assessment *CSAB*<br><br>AIM 1 & AIM 2: Research and Logistical Process *Translations Spanish live*	CSAB	Pre-implementation (AIM 1)	Based on feedback or suggestions	To enhance the impact or success of the intervention for all or important subgroups (effectiveness)<br><br>To make it possible to involve more teams, team members or staff (adoption)<br><br>For practical reasons (implementation)	Number or type of patients engaged reached- No change<br><br>Participation of teams or staff- No change<br><br>Reimbursement or financial implications for the practice – No change<br><br>Efficiency-No change<br><br>Meaningful engagement- Increased
Gift Card Change for CSAB members	Types of gift cards provided and how they are given to CSAB members and how they are documented is streamlined	Planned	How the intervention/ program is presented/ delivered - how core components are operationalized	Condensing a component	AIM 1: Partner Engagement and Community Needs/Assets Assessment *CSAB*	CSAB	Pre-implementation (AIM 1)	Based on financial incentives/ payment	To increase the number or type of patients contacted (reach)<br><br>To save money or other resources (implementation)	Number or type of patients engaged reached- No change<br><br>Participation of teams or staff- No change<br><br>Reimbursement or financial implications for the practice – No change<br><br>Efficiency-No change

										Meaningful engagement-Increased
CSAB Stakeholder engagement Survey Edits	A comment box was added for additional feedback to stakeholder engagement survey.	Planned	The format	Adding a component	AIM 1: Partner Engagement and Community Needs/Assets Assessment *CSAB*	CSAB	Pre-implementation (AIM 1)	Based on feedback or suggestions	To enhance the impact or success of the intervention for all or important subgroups (effectiveness)<br><br>To make it possible to involve more teams, team members or staff (adoption)	Number or type of patients engaged reached- No change<br><br>Participation of teams or staff- No change<br><br>Reimbursement or financial implications for the practice – No change<br><br>Efficiency-No change<br><br>Meaningful engagement-Increased
Stakeholder Survey Edits	Stakeholder survey was edited to include a "Not Applicable" option for items	Planned	The format	Adding a component	AIM 1: Partner Engagement and Community Needs/Assets Assessment *CSAB*	CSAB	Pre-implementation (AIM 1)	Based on feedback or suggestions	To enhance the impact or success of the intervention for all or important subgroups (effectiveness)	Number or type of patients engaged reached- No change<br><br>Participation of teams or staff- No change<br><br>Reimbursement or financial implications for the practice – No change<br><br>Efficiency-No change<br><br>Meaningful engagement-Increased
Change in order of presenters	Community members invited to speak first	Planned	The format<br><br>Personnel involved	Tailoring to individuals<br><br>Extending a component	AIM 1: Partner Engagement and Community Needs/Assets Assessment *CSAB*	CSAB	Pre-implementation (AIM 1)	Based on feedback or suggestions	To increase the number or type of patients contacted (reach)	Number or type of patients engaged reached- No change

									To enhance the impact or success of the intervention for all or important subgroups (effectiveness)	Participation of teams or staff- No change<br><br>Reimbursement or financial implications for the practice – No change<br><br>Efficiency-No change<br><br>Meaningful engagement- Increased
Change name from observer to documenter	Name changed from observer to documenter to recognize specific role as more engaged and not just someone who is watching	Planned	The setting<br><br>Personnel involved	Tailoring to individuals<br><br>Substituting for a component	AIM 1: Partner Engagement and Community Needs/Assets Assessment *CSAB*	CSAB	Pre-implementation (AIM 1)	Based on our vision or values<br><br>Based on feedback or suggestions	To enhance the impact or success of the intervention for all or important subgroups (effectiveness)<br><br>To make it possible to involve more teams, team members or staff (adoption)<br><br>To respond to external pressures or policy	Number or type of patients engaged reached- No change<br><br>Participation of teams or staff- No change<br><br>Reimbursement or financial implications for the practice – No change<br><br>Efficiency-No change<br><br>Meaningful engagement- Increased
Added Break to Meetings	Added break to meetings to allow interpreters opportunity to take a break	Planned	The setting<br><br>The format	Adding a component	AIM 1: Partner Engagement and Community Needs/Assets Assessment *CSAB*	CSAB	Pre-implementation (AIM 1)	Based on feedback or suggestions	To enhance the impact or success of the intervention for all or important subgroups (effectiveness)<br><br>To institutionalize or sustain the intervention (maintenance)	Number or type of patients engaged reached- No change<br><br>Participation of teams or staff- No change<br><br>Reimbursement or financial implications for the practice – No change<br><br>Efficiency-No change

										Meaningful engagement-Increased
Meeting dates changed	Meeting dates changed based on feedback from members. Date changes were made in order to recognize community events or religious holidays	Planned	The setting	Substituting for a component	AIM 1: Partner Engagement and Community Needs/Assets Assessment *CSAB*	CSAB	Pre-implementation (AIM 1)	Based on feedback or suggestions	For practical reasons (implementation)<br><br>To respond to external pressures or policy	Number or type of patients engaged reached-Increased<br><br>Participation of teams or staff-No change<br><br>Reimbursement or financial implications for the practice – No change<br><br>Efficiency-No change<br><br>Meaningful engagement-Increased
Recognition of Need for More Bilingual Staff	After a few months working at MCHC completing AIM 1 and starting AIM 2, it became evident that the need for all staff to be bilingual was necessary. Bilingual interns recruited to help with onsite testing. New CRC posting submitted to HR with bilingual requirement	Planned	Personnel involved<br><br>How the intervention/ program is presented/ delivered - how core components are operationalized	Substituting for a component	AIM 2: COVID-19 Testing	CSAB	Early Implementation (AIM 2)	Based on feedback or suggestions	To increase the number or type of patients contacted (reach)<br><br>To enhance the impact or success of the intervention for all or important subgroups (effectiveness)<br><br>To deliver intervention more consistently; better for practice, patient flow or EHR (implementation)<br><br>For practical reasons (implementation)	Number or type of patients engaged reached-Increased<br><br>Effectiveness-Increased<br><br>Participation of teams or staff-No change<br><br>Implementation - No change<br><br>Maintenance-Increased<br><br>Reimbursement or financial implications for the practice – Increased

										Efficiency – No change<br><br>Meaningful engagement- Increased
REDCap Multilingual Tool Needs VPN	REDCap multilingual tool needs to be on a UCSD vpn in order to work on tablets/computers/phone. Participants cannot connect to vpn on phones so they cannot complete survey on phone. All testing tablets must be connected to VPN at the start of the day.	Planned	How the intervention/ program is presented/ delivered - how core components are operationalized	Removing a component	AIM 2: COVID-19 Testing	Entire or most of the team	Early Implementation (AIM 2)	Based on pragmatic/ practical considerations (i.e., is this the only way it could work?)<br><br>Based on feedback or suggestions	To respond to external pressures or policy	Number or type of patients engaged reached- Increased<br><br>Effectiveness- No change<br><br>Participation of teams or staff- No change<br><br>Implementation - No change<br><br>Maintenance- No change<br><br>Reimbursement or financial implications for the practice – Increased<br><br>Efficiency – Increased<br><br>Meaningful engagement- Increased

Preferred Method of Contact for ROR	Preferred method of contact has been changed to reflect both phone and text as an option along with email for ROR. Distinction between phone and text was important for those who do not have text option.	Planned	How the intervention/program is presented/delivered - how core components are operationalized	Extending a component	AIM 2: COVID-19 Testing	CRC<br><br>Other: REDCap Administrator	Early Implementation (AIM 2)	Based on pragmatic/ practical considerations (i.e. is this the only way it could work?)	To increase the number or type of patients contacted (reach)<br><br>For practical reasons (implementation)	Number or type of patients engaged reached- No change<br><br>Effectiveness- Increased<br><br>Participation of teams or staff- No change<br><br>Implementation - Increased<br><br>Maintenance- No change<br><br>Reimbursement or financial implications for the practice – No change<br><br>Efficiency – Increased<br><br>Meaningful engagement- Increased
Condensing Cohort Consents	Cohort consents were condensed to simplify consenting process. IRB amendment going from larger number of consent forms to smaller, streamlined number of consents.	Planned	The format<br><br>How the intervention/ program is presented/ delivered - how core components are operationalized	Condensing a component	AIM 2: COVID-19 Testing	CRC	Early Implementation (AIM 2)	Based on feedback or suggestions	To deliver intervention more consistently; better for practice, patient flow or EHR (implementation)<br><br>For practical reasons (implementation)	Number or type of patients engaged reached- No change<br><br>Effectiveness- No change<br><br>Participation of teams or staff- No change<br><br>Implementation - No change<br><br>Maintenance- No change<br><br>Reimbursement or financial implications for the practice – No change<br><br>Efficiency- Increased

										Meaningful engagement- No change
Revision of REDCap Forms for Cohort Identificatio n	Inclusion of cohort identification added into REDCap initial survey for participants to self-identify cohort he/she fit into. This compliments the condensing of consent forms.	Planned	The format<br><br>How the intervention/ program is presented/ delivered - how core components are operationalized	Extending a component	AIM 2: COVID-19 Testing	CRC	Early Implementation (AIM 2)	Based on pragmatic/ practical considerations (i.e., is this the only way it could work?)<br><br>Based on feedback or suggestions	To enhance the impact or success of the intervention for all or important subgroups (effectiveness)<br><br>To deliver intervention more consistently; better for practice, patient flow or EHR (implementation)	Number or type of patients engaged reached- No change<br><br>Effectiveness- No change<br><br>Participation of teams or staff- No change<br><br>Implementation - No change<br><br>Maintenance- No change<br><br>Reimbursement or financial implications for the practice – No change<br><br>Efficiency- Increased<br><br>Meaningful engagement- No change

Move from NextGen to Inspect Forms	Documentation of participants and lab orders have been moved from NextGen to Inspect (Excite Lab) forms to improve workflow speed and participant processing while on site.	Planned	How the intervention/ program is presented/ delivered - how core components are operationalized	Substituting for a component	AIM 2: COVID-19 Testing	CRC	Early Implementation (AIM 2)	Based on our knowledge or experience of working with patients<br><br>Based on pragmatic/ practical considerations (i.e., is this the only way it could work?)<br><br>Based on feedback or suggestions	To increase the number or type of patients contacted (reach)<br><br>To enhance the impact or success of the intervention for all or important subgroups (effectiveness)<br><br>To make it possible to involve more teams, team members or staff (adoption)<br><br>For practical reasons (implementation)	Number or type of patients engaged reached-Increased<br><br>Effectiveness-Increased<br><br>Participation of teams or staff-Increased<br><br>Implementation - Increased<br><br>Maintenance-Increased<br><br>Reimbursement or financial implications for the practice – No change<br><br>Efficiency-Increased<br><br>Meaningful engagement-Increased
Flyer Update	Flyers were updated and IRB approved to allow for address changes to be made based on location of mobile unit. Able to make address changes without having to make an amendment every time.	Planned	The format	Extending a component	AIM 2: COVID-19 Testing	CRC	Early Implementation (AIM 2)	Based on pragmatic/ practical considerations (i.e., is this the only way it could work?)<br><br>Based on feedback or suggestions	To increase the number or type of patients contacted (reach)<br><br>To enhance the impact or success of the intervention for all or important subgroups (effectiveness)<br><br>To make it possible to involve more teams, team members or staff (adoption)<br><br>To deliver intervention more consistently; better for practice, patient	Number or type of patients engaged reached-Increased<br><br>Effectiveness-Increased<br><br>Participation of teams or staff-No change<br><br>Implementation - Increased<br><br>Maintenance-Increased<br><br>Reimbursement or financial implications for the practice – No change<br><br>Efficiency-Increased

									flow or EHR (implementation)<br><br>For practical reasons (implementation)<br><br>To institutionalize or sustain the intervention (maintenance)	Meaningful engagement-Increased
SYH Employee not testing as part of CO-CREATE	SYH Employees asked not to get tested through CO-CREATE to protect patient testing time and staff, and prevent further staff burn out during Omicron surge.	Planned	How the intervention/ program is presented/ delivered - how core components are operationalized	Removing a component	AIM 2: COVID-19 Testing	CRC	Implementation (AIM 2)	Based on pragmatic/practical considerations (i.e., is this the only way it could work?)<br><br>Based on feedback or suggestions	To respond to external pressures or policy	Number or type of patients engaged reached-Increased<br><br>Effectiveness-Increased<br><br>Participation of teams or staff-No change<br><br>Implementation - No change<br><br>Maintenance-Increased<br><br>Reimbursement or financial implications for the practice – No change<br><br>Efficiency-Increased<br><br>Meaningful engagement-Increased

RAPID Test Home-Kits	Patients are able to come on-site to pick-up free antigen test home-kits to take home. Patients are able to get as many boxes as they need (each box contains two testing kits). These kits are specifically for patients who have tested positive in the last 90 days and need proof of a negative test result.	Planned	The format	Adding a component	AIM 2: COVID-19 Testing	SYH Research Staff	Late Implementation (AIM 2)	Based on pragmatic/practical considerations (i.e., is this the only way it could work?)<br><br>Based on feedback or suggestions	To institutionalize or sustain the intervention (maintenance)	Number or type of patients engaged reached- Increased<br><br>Effectiveness- Increased<br><br>Participation of teams or staff- No change<br><br>Implementation - Increased<br><br>Maintenance- No change<br><br>Reimbursement or financial implications for the practice – No change<br><br>Efficiency- Increased<br><br>Meaningful engagement- Increased
Data Reporting on CO-CREATE website	Data reporting on website: data that aligns w/project AIMs (display data captured from survey responses # of tests, # of positives, #vaccines received, barriers to accessing testing, barriers to accessing vaccine #affected by covid-19, # +covid test, where get info from; comorbidities) was added to CO-CREATE website.	Planned	How the intervention/ program is presented/ delivered - how core components are operationalized	Tailoring to individuals	AIM 1 & AIM 2: Research and Logistical Process *Personnel*	Entire or most of team	Implementation (AIM 2)	Based on financial incentives/ payment<br><br>Based on feedback or suggestions	To institutionalize or sustain the intervention (maintenance)	N/a

Return of Result After testing flyer	Return of results flyer created by Luis w/information on when to expect results and CO-CREATE contact information.	Planned	How the intervention/ program is presented/ delivered - how core components are operationalized	Adding a component	AIM 2: COVID-19 Testing	Entire or most of team<br><br>CSAB	Implementation (AIM 2)	Based on our vision or values	To institutionalize or sustain the intervention (maintenance)	Number or type of patients engaged reached- No change<br><br>Effectiveness- No change<br><br>Participation of teams or staff- No change<br><br>Implementation - Increased<br><br>Maintenance- No change<br><br>Reimbursement or financial implications for the practice – No change<br><br>Efficiency- Increased<br><br>Meaningful engagement- Increased
EXCITE PILOT Program	Participants asked to complete two separate COVID tests for EXCITE. Onsite affects, slower line through testing. Abel to opt out.	Planned	How the intervention/ program is presented/ delivered - how core components are operationalized	Extending a component	AIM 2: COVID-19 Testing	SYH Research Staff	Implementation (AIM 2)	Based on our knowledge or experience of working with patients	To enhance the impact or success of the intervention for all or important subgroups (effectiveness)	Number or type of patients engaged reached- No change<br><br>Effectiveness- No change<br><br>Participation of teams or staff- No change<br><br>Implementation - No change<br><br>Maintenance- No change<br><br>Reimbursement or financial implications for the practice – No change<br><br>Efficiency- Decreased

										Meaningful engagement- Increased
Additional Registration Questions	There were added questions to on-site Testing excel that are asked during registration of participants: symptom and vaccination status, and how they hear about testing site.	Planned	How the intervention/ program is presented/ delivered - how core components are operationalized	Extending a component	AIM 2: COVID-19 Testing	Principle or Co-Principle Investigator(s)	Implementation (AIM 2)	Other: Research team trying to establish effectiveness of different testing methods	For practical reasons (implementation)	Number or type of patients engaged reached- No change<br><br>Effectiveness- No change<br><br>Participation of teams or staff- No change<br><br>Implementation - No change<br><br>Maintenance- No change<br><br>Reimbursement or financial implications for the practice – No change<br><br>Efficiency- No change<br><br>Meaningful engagement- No change

SASEA Responsive Testing	CO-CREATE assisted with responsive testing for SASEA's waste water project with SYSD.	Planned	How the intervention/ program is presented/ delivered - how core components are operationalized	Adding a component<br><br>Integrating with other programs we are doing	AIM 2: COVID-19 Testing	Entire or most of team	Implementation (AIM 2)	Based on pragmatic/practical considerations (i.e., is this the only way it could work?)	To increase the number or type of patients contacted (reach)<br><br>To enhance the impact or success of the intervention for all or important subgroups (effectiveness)	Number or type of patients engaged reached- Increased<br><br>Effectiveness- Increased<br><br>Implementation - No change<br><br>Maintenance- Decreased<br><br>Reimbursement or financial implications for the practice – Increased<br><br>Efficiency- Decreased<br><br>Meaningful engagement- Decreased
Arm 1 participant extension	IBR approval to increase Arm 1 testing from 15 to 60 visits through out the study period.	Planned	How the intervention/ program is presented/ delivered - how core components are operationalized	Extending a component	AIM 1 & AIM 2: Research and Logistical Process<br>*IRB*	CRC<br><br>Principle or Co-Principle Investigator(s)	Implementation (AIM 2)	Based on our vision or values	To institutionalize or sustain the intervention (maintenance)	Number or type of patients engaged reached- Increased<br><br>Effectiveness- Increased<br><br>Participation of teams or staff- Increased<br><br>Implementation - No change<br><br>Maintenance- Increased<br><br>Reimbursement or financial implications for the practice – Increased<br><br>Efficiency- No change<br><br>Meaningful engagement- No change

Electronic gift cards	Changing to electronic Amazon gift cards for survey compensation. Made change because we were out of Walmart, waiting for more to come in.	Planned	The format	Substituting for a component	AIM 2: COVID-19 Testing	CRC<br><br>Principle or Co-Principle Investigator(s)	Implementation (AIM 2)	Based on our vision or values	For practical reasons (implementation)	Number or type of patients engaged reached- No change<br><br>Effectiveness- No change<br><br>Participation of teams or staff- No change<br><br>Implementation - No change<br><br>Maintenance- No change<br><br>Reimbursement or financial implications for the practice – No change<br><br>Efficiency- Increased<br><br>Meaningful engagement- No change
Condensing of consenting station	Instead of three stations, there are two: registration/consent and testing. Participants are now asked if they would like to participate in the survey at registration where they are consented.	Planned	How the intervention/ program is presented/ delivered - how core components are operationalized	Condensing a component	AIM 2: COVID-19 Testing	CRC<br><br>SYH Research Staff	Implementation (AIM 2)	Based on pragmatic/ practical considerations (i.e., is this the only way it could work?)	For practical reasons (implementation)	Number or type of patients engaged reached- Increased<br><br>Effectiveness- Increased<br><br>Participation of teams or staff- Increased<br><br>Implementation - No change<br><br>Maintenance- Increased<br><br>Reimbursement or financial implications for the practice – No change<br><br>Efficiency- Increase

										Meaningful engagement- No change
Omicron Surge on-site hours	On site hours changed to complete all testing by normal testing hours and to accommodate for our daily testing capacity during Omicron surge. Mon-Thur the line is closed at 12:30pm and Fri 10:15am.	Unplanned	The format	Tailoring to individuals	AIM 2: COVID-19 Testing	CRC<br><br>SYH Research Staff	Implementation (AIM 2)	Based on pragmatic/ practical considerations (i.e., is this the only way it could work?)	To deliver intervention more consistently; better for practice, patient flow or EHR (implementation)	Number or type of patients engaged reached- Decreased<br><br>Effectiveness- Decreased<br><br>Participation of teams or staff- Decreased<br><br>Implementation - No change<br><br>Maintenance- Increased<br><br>Reimbursement or financial implications for the practice – No change<br><br>Efficiency- Increased<br><br>Meaningful engagement- No change

CSAB Meeting Changed	CSAB meeting in April changed to 2nd Wednesday of month (Apr 13) to accommodate the need to address time-sensitive topics related to the testing program	Planned	The setting	Changing the order of components	AIM 1: Partner Engagement and Community Needs/Assets Assessment *CSAB*	Entire or most of team	Late Implementation (AIM 2)	Based on pragmatic/ practical considerations (i.e., is this the only way it could work?)	To enhance the impact or success of the intervention for all or important subgroups (effectiveness)	Reimbursement or financial implications for the practice – Decreased<br><br>Meaningful engagement- No change
Global ARC Meeting Change	Change made to weekly Global ARC meeting schedule. Move to 11am PT effective March 7, 2022	Planned	The setting	Tailoring to individuals	Other: Global ARC Meeting/ Staff	Entire or most of team	Late Implementation (AIM 2)	Based on feedback or suggestions	To make it possible to involve more teams, team members or staff (adoption)	N/A

Billboards	Billboards to represent community with SYH staff and community members and the Billboards were presented during a CSAB meeting.	Planned	Other: advertisement of the program from using flyers to promote the program to using a larger scale in order to reach more people	Extending a component	AIM 1: Partner Engagement and Community Needs/Assets Assessment<br>*CSAB*<br>*Other: Community Outreach*	Other: UCSD & Global ARC Staff	Implementation (AIM 2)	Based on pragmatic/ practical considerations (i.e., is this the only way it could work?)	To increase the number or type of patients contacted (reach)	Number or type of patients engaged reached- No change<br><br>Effectiveness- No change<br><br>Participation of teams or staff- No change<br><br>Implementation - No change<br><br>Maintenance- No change<br><br>Reimbursement or financial implications for the practice – Increased<br><br>Efficiency- No change<br><br>Meaningful engagement- No change
Survey Email Distribution	The primary form of survey distribution is via email. If participants chose to take part in the survey they are sent a link to their email at the end of the day. If participants do not have an e-mail, they are given a paper survey to complete at home.	Unplanned	The format	Adding a component	COVID-19 Testing	Entire or most of team	Implementation (AIM 2)	Based on financial incentives/ payment<br><br>Based on feedback or suggestions	For practical reasons (implementation)	Number or type of patients engaged reached- No change<br><br>Effectiveness- No change<br><br>Participation of teams or staff- No change<br><br>Implementation - No change<br><br>Maintenance- No change<br><br>Reimbursement or financial implications for the practice – Increased<br><br>Efficiency- Increased

										Meaningful engagement- No change
Return of Results - Double Check	A coordinator is assigned a remote workday for return of results. Before any results are sent a second coordinator must double check the Testing log to ensure participant name matches with their email.	Planned	How the intervention/ program is presented/ delivered - how core components are operationalized	Adding a component	COVID-19 Testing	CRC<br><br>SYH Research Staff	Late Implementation (AIM 2)	Based on pragmatic/ practical considerations (i.e., is this the only way it could work?)<br><br>Based on financial incentives/ payment	To enhance the impact or success of the intervention for all or important subgroups (effectiveness)<br><br>To deliver intervention more consistently; better for practice, patient flow or EHR (implementation)<br><br>For practical reasons (implementation) To institutionalize or sustain the intervention (maintenance)<br><br>To respond to external pressures or policy	Number or type of patients engaged reached- No change<br><br>Effectiveness- No change<br><br>Participation of teams or staff- No change<br><br>Implementation - Increased<br><br>Maintenance- No change<br><br>Reimbursement or financial implications for the practice – Increased<br><br>Efficiency- No change<br><br>Meaningful engagement- No change

Return of Positive Results- Omicron Surge	SYH RN's are available to help make calls for positive SYH patients. To identify the SYH patients a column to the SYH Result Excel was added where the on-call coordinator through a VLOOKUP pulls the SYH patient status from the on-site testing excel Coordinators are able to return positive results and must ask participants if they would like an additional consult from the study doctor/nurse.	Planned	Personnel involved<br><br>How the intervention/ program is presented/ delivered - how core components are operationalized	Adding a component	COVID-19 Testing	CRC	Late Implementation (AIM 2)	Based on pragmatic/ practical considerations (i.e., is this the only way it could work?)	To increase the number or type of patients contacted (reach)<br><br>To enhance the impact or success of the intervention for all or important subgroups (effectiveness)<br><br>To make it possible to involve more teams, team members or staff (adoption)<br>To deliver intervention more consistently; better for practice, patient flow or EHR (implementation)<br><br>For practical reasons (implementation)<br><br>To institutionalize or sustain the intervention (maintenance)<br><br>To respond to external pressures or policy	Number or type of patients engaged reached- No change<br><br>Effectiveness- No change<br><br>Participation of teams or staff- No change<br><br>Implementation - Increased<br><br>Maintenance- Increased<br><br>Reimbursement or financial implications for the practice – No change<br><br>Efficiency- Increased<br><br>Meaningful engagement- Increased

Result Report/ Testing Details	Staff are able to use Vlookup and data export to upload Testing Details onto RedCap after receiving test results for participants. This was due to the Omicron Surge and staff falling behind on uploading testing details.	Unplanned	The setting	Substituting for a component	COVID-19 Testing	Entire of most of team<br><br>CRC	Late Implementation (AIM 2)	Based on pragmatic/ practical considerations (i.e., is this the only way it could work?)	To deliver intervention more consistently; better for practice, patient flow or EHR (implementation)<br><br>For practical reasons (implementation)<br><br>To respond to external pressures or policy	Number or type of patients engaged reached- No change<br><br>Effectiveness- No change<br><br>Participation of teams or staff- No change<br><br>Implementation - No change<br><br>Maintenance- No change<br><br>Reimbursement or financial implications for the practice – No change<br><br>Efficiency- Increased<br><br>Meaningful engagement- No change
Electronic gift cards	Gift cards for participating in initial and return survey are sent as electronic Amazon gift card to the participants email.	Unplanned	The setting	Adding a component	COVID-19 Testing	CRC<br><br>SYH Research Staff	Implementation (AIM 2)	Based on our knowledge or experience of working with patients<br><br>Based on financial incentives/ payment<br><br>Based on feedback or suggestions	For practical reasons (implementation)	Number or type of patients engaged reached- No change<br><br>Effectiveness- No change<br><br>Participation of teams or staff- No change<br><br>Implementation - No change<br><br>Maintenance- No change<br><br>Reimbursement or financial implications for the practice – No change<br><br>Efficiency- No change

										Meaningful engagement- No change
Return of Results- Nurse on Call	A UCSD nurse is now available for return of results.	Planned	Personnel involved	Adding a component	AIM 1 & AIM 2: Research and Logistical Process	CRC<br><br>SYH Research Staff	Late Implementation (AIM 2)	Based on pragmatic/ practical considerations (i.e., is this the only way it could work?)<br><br>Based on financial incentives/ payment<br><br>Based on feedback or suggestions	To increase the number or type of patients contacted (reach)<br><br>To enhance the impact or success of the intervention for all or important subgroups (effectiveness)<br><br>To make it possible to involve more teams, team members or staff (adoption)<br><br>To deliver intervention more consistently; better for practice, patient flow or EHR (implementation)	Number or type of patients engaged reached- No change<br><br>Effectiveness- No change<br><br>Participation of teams or staff- No change<br><br>Implementation - Increased<br><br>Maintenance- No change<br><br>Reimbursement or financial implications for the practice – Increased<br><br>Efficiency- Increased<br><br>Meaningful engagement- No change

IRB Amendment to add RAPID Testing	IRB Amendment to add RAPID testing on-site. Currently, patients are able to take a antigen test-kit home but staff are unable to provide guidance when using the kit. This is due to the high need of RAPID test for patients who have recently tested positive and need to return to work.	Planned	How the intervention/program is presented/delivered - how core components are operationalized	Tailoring to individuals<br><br>Adding a component	AIM 2: COVID-19 Testing<br><br>AIM 1 & AIM 2: Research and Logistical Process *Personnel*	Principle or Co-Principle Investigator(s)	Late Implementation (AIM 2)	Based on pragmatic/ practical considerations (i.e., is this the only way it could work?)<br><br>Based on financial incentives/ payment	To increase the number or type of patients contacted (reach)<br><br>To enhance the impact or success of the intervention for all or important subgroups (effectiveness)<br><br>To deliver intervention more consistently; better for practice, patient flow or EHR (implementation)<br><br>To institutionalize or sustain the intervention (maintenance)<br><br>To respond to external pressures or policy	Number or type of patients engaged reached- No change<br><br>Effectiveness- No change<br><br>Participation of teams or staff- No change<br><br>Implementation - No change<br><br>Maintenance- No change<br><br>Reimbursement or financial implications for the practice – No change<br><br>Efficiency- No change<br><br>Meaningful engagement- No change
Survey Distribution	During the Omicron Surge staff were sending out surveys as mass emails via REDCap instead of sending individual surveys and they were distributing paper surveys as well. As of April, staff have returned to sending out surveys once the profile has been created to avoid confusion on which participants have received paper surveys and those who have	Planned	The setting	Tailoring to individuals	AIM 2: COVID-19 Testing *IRB*	Entire or most of team	Late Implementation (AIM 2)	Based on feedback or suggestions	To enhance the impact or success of the intervention for all or important subgroups (effectiveness)<br><br>For practical reasons (implementation)<br><br>To save money or other resources (implementation)	Number or type of patients engaged reached- No change<br><br>Effectiveness- No change<br><br>Participation of teams or staff- No change<br><br>Implementation - No change<br><br>Maintenance- No change<br><br>Reimbursement or financial implications for the practice – No change

	selected receiving an Amazon gift card with those who have opted out.									Efficiency- Increased<br><br>Meaningful engagement- No change
SYH Patient Report Disclaimer	A disclaimer has been added to SYH patient report stating that Patients have been contacted. The following provides an idea of the disclaimers added:<br>Negative- confirming patients were notified of results<br>Positive- patient contacted with their result.	Planned	How the intervention/program is presented/delivered - how core components are operationalized	Tailoring to individuals<br><br>Adding a component	AIM 2: COVID-19 Testing	CRC<br><br>SYH Research Staff	Late Implementation (AIM 2)	Based on feedback or suggestions	To enhance the impact or success of the intervention for all or important subgroups (effectiveness)<br><br>For practical reasons (implementation)<br><br>To respond to external pressures or policy	N/A

REDCap SYH Stauts	SYH Status has been added to testing details for all REDCap IDs to distinguish which participants are being seen by SYH.	Planned	Other: REDCap changes	Adding a component	AIM 2: COVID-19 Testing	Entire or most of team	Late Implementation (AIM 2)	Based on our vision	To respond to external pressures or policy	Number or type of patients engaged reached- No change<br><br>Effectiveness- No change<br><br>Participation of teams or staff- No change<br><br>Implementation - No change<br><br>Maintenance- No change<br><br>Reimbursement or financial implications for the practice – No change<br><br>Efficiency- Increased<br><br>Meaningful engagement- No change
Church of Christ Event	CO-CREATE was able to offer testing and surveys at a community event during a Saturday, which allowed the team to work with a diverse population. The team was also given the opportunity to flyer and meet other organizations in San Diego.	Planned	The setting<br><br>Target population	Tailoring to individuals	AIM 2: COVID-19 Testing	CRC<br><br>SYH Investigator(s)	Late Implementation (AIM 2)	Based on our vision<br><br>Based on feedback or suggestions	To increase the number or type of patients contacted (reach)<br><br>o make it possible to involve more teams, team members or staff (adoption)	Number or type of patients engaged reached- Increased<br><br>Effectiveness- Increased<br><br>Participation of teams or staff- No change<br><br>Implementation - No change<br><br>Maintenance- No change<br><br>Reimbursement or financial implications for the practice – Increased<br><br>Efficiency- No change

										Meaningful engagement-Increased
Collaboration with UPAC	On March 29, CO-CREATE CRC's met with UPAC to collaborate on future events in the Southeast San Diego region. We presented to them about our program and finalized dates for future events.	Planned	Target population	Tailoring to individuals<br><br>Adding a component	AIM 2: COVID-19 Testing	CRC<br><br>SYH Research Staff	Late Implementation (AIM 2)	Based on our knowledge or experience of working with patients<br><br>Based on QI data, summary information or results	To increase the number or type of patients contacted (reach)	Number or type of patients engaged reached-Increased<br><br>Effectiveness-Increased<br><br>Participation of teams or staff-No change<br><br>Implementation - No change<br><br>Maintenance-No change<br><br>Reimbursement or financial implications for the practice – Increased<br><br>Efficiency- No change<br><br>Meaningful engagement-Increased

Re-testing Educational Information	Patients who are positive have shifted from asking vaccine-oriented questions to retesting question. As a result, the team has developed testing educational information and guidelines about retesting when positive to help ease convalescent cases.	Planned	The format	Tailoring to individuals	AIM 2: COVID-19 Testing	Entire or most of team	Late Implementation (AIM 2)	Based on feedback or suggestions	To enhance the impact or success of the intervention for all or important subgroups (effectiveness)<br><br>To deliver intervention more consistently; better for practice, patient flow or EHR (implementation)	Number or type of patients engaged reached- No change<br><br>Effectiveness- No change<br><br>Participation of teams or staff- No change<br><br>Implementation - No change<br><br>Maintenance- No change<br><br>Reimbursement or financial implications for the practice – No change<br><br>Efficiency- Increased<br><br>Meaningful engagement- Increased
Testing Hours	Increased testing days to five times a week. Monday-Thursday 8:30-3:00pm and Friday 9-12pm.	Planned	The format	Extending a component	AIM 2: COVID-19 Testing	Entire or most of team	Implementation (AIM 2)	Based on pragmatic/ practical considerations (i.e., is this the only way it could work?)	To increase the number or type of patients contacted (reach)	Number or type of patients engaged reached- Increased<br><br>Effectiveness- Increased<br><br>Participation of teams or staff- Increased<br><br>Implementation - Increased<br><br>Maintenance- Increased<br><br>Reimbursement or financial implications for the practice – Increased<br><br>Efficiency- Increased

										Meaningful engagement- Increased
Global ARC Meeting Time Change	Changes to weekly Global ARC meeting schedule. Moved to 12pm effective Sep 27th. Except 2nd Monday/month move to 8:30am	Planned	Other: time of meeting	Tailoring to individuals	Other: Partner Engagement	Entire or most of team	Implementation (AIM 2)	Based on our knowledge or experience of working with patients<br><br>Based on pragmatic/ practical considerations (i.e., is this the only way it could work?)	To make it possible to involve more teams, team members or staff (adoption)	N/a

Registering Participants	Before staff were registering participants as SYH patients, then they transitioned to registering participants on an Excel sheet.	Planned	The format	Tailoring to individuals	AIM 2: COVID-19 Testing	Other: Global ARC	Implementation (AIM 2)	Based on our vision or values<br><br>Based on our knowledge or experience of working with patients<br><br>Based on feedback or suggestions	To enhance the impact or success of the intervention for all or important subgroups (effectiveness)<br><br>For practical reasons (implementation)	Number or type of patients engaged reached- Increased<br><br>Effectiveness- Increased<br><br>Participation of teams or staff- Increased<br><br>Implementation - Increased<br><br>Maintenance- Increased<br><br>Efficiency- Increased<br><br>Meaningful engagement- No change
Informational Flyers	No informational flyers were provided on-site, only on social media, the team transitioned into creating Informational flyers to provide on-site to promote awareness on the importance of testing and vaccinations.	Planned	The format	Tailoring to individuals<br><br>Adding a component	AIM 2: COVID-19 Testing	CRC	Implementation (AIM 2)	Based on our knowledge or experience of working with patients<br><br>Based on QI data, summary information or results<br><br>Based on feedback or suggestions	To increase the number or type of patients contacted (reach)<br><br>To enhance the impact or success of the intervention for all or important subgroups (effectiveness)<br><br>To institutionalize or sustain the intervention (maintenance)	Number or type of patients engaged reached- Increased<br><br>Effectiveness- Increased<br><br>Participation of teams or staff- No change<br><br>Implementation - Increased<br><br>Maintenance- Increased<br><br>Reimbursement or financial implications for the practice – No change<br><br>Efficiency- No change<br><br>Meaningful engagement- Increased

PPE	Staff were only wearing masks and gloves, shortly after they began to use face shields, goggles, and gowns.	Planned	The format	Adding a component	AIM 2: COVID-19 Testing	Entire or most of team	Implementation (AIM 2)	Based on pragmatic/ practical considerations (i.e., is this the only way it could work?)<br><br>Based on feedback or suggestions	To deliver intervention more consistently; better for practice, patient flow or EHR (implementation)<br><br>To respond to external pressures or policy	Reimbursement or financial implications for the practice – Increased
On-Site Location	CO-CREATE was stationed in front of the clinic within a three space parking lot. The team then expanded the testing space to encourage social distancing and to increase our capacity for patients.	Planned	The setting	Tailoring to individuals	AIM 2: COVID-19 Testing	Entire or most of team	Early Implementation (AIM 2)	Based on pragmatic/ practical considerations (i.e., is this the only way it could work?)<br><br>Based on feedback or suggestions	To increase the number or type of patients contacted (reach)<br><br>To enhance the impact or success of the intervention for all or important subgroups (effectiveness)<br><br>To make it possible to involve more teams, team members or staff (adoption)<br><br>To deliver intervention more consistently; better for practice, patient flow or EHR (implementation)<br><br>For practical reasons (implementation)	Number or type of patients engaged reached-Increased<br><br>Effectiveness-Increased<br><br>Participation of teams or staff-No change<br><br>Implementation - No change<br><br>Maintenance-No change<br><br>Reimbursement or financial implications for the practice – No change<br><br>Efficiency-Increased<br><br>Meaningful engagement-No change

									To institutionalize or sustain the intervention (maintenance)	
Staff	We had a decrease in staff that were present on-site from both UCSD and SYH. Interns and a new staff from SYH began supporting us on-site.	Planned	Personnel involved	Adding a component	AIM 2: COVID-19 Testing	Entire or most of team	Implementation (AIM 2)	Based on our vision or values<br><br>Based on our knowledge or experience of working with patients<br><br>Based on pragmatic/ practical considerations (i.e., is this the only way it could work?)<br><br>Based on feedback or suggestions	To enhance the impact or success of the intervention for all or important subgroups (effectiveness)<br><br>To make it possible to involve more teams, team members or staff (adoption)	Number or type of patients engaged reached- Increased<br><br>Effectiveness- Increased<br><br>Participation of teams or staff- Increased<br><br>Implementation - Increased<br><br>Maintenance- Increased<br><br>Reimbursement or financial implications for the practice – Increased<br><br>Efficiency- Increased<br><br>Meaningful engagement- Increased

Social Distancing Awareness	Before the team would verbally inform patients about social distancing awareness, the team then began to use caution tape, 6 foot markers, and cones to promote safe testing between patients and staff.	Planned	The setting	Adding a component	AIM 2: COVID-19 Testing	Entire or most of team	Implementation (AIM 2)	Based on pragmatic/ practical considerations (i.e., is this the only way it could work?)<br><br>Based on feedback or suggestions	To enhance the impact or success of the intervention for all or important subgroups (effectiveness)<br><br>To deliver intervention more consistently; better for practice, patient flow or EHR (implementation)	Reimbursement or financial implications for the practice – Increased
New UCSD Providers	The team on boarded three new providers to the UCSD to help with the increase in positive COVID cases (return of results).	Planned	How the intervention/program is presented/delivered - how core components are operationalized	Adding a component	AIM 2: COVID-19 Testing	Entire or most of team	Late Implementation (AIM 2)	Based on pragmatic/ practical considerations (i.e., is this the only way it could work?)<br><br>Based on feedback or suggestions	To increase the number or type of patients contacted (reach)<br><br>To enhance the impact or success of the intervention for all or important subgroups (effectiveness)	Number or type of patients engaged reached-Increased<br><br>Effectiveness-No change<br><br>Participation of teams or staff-No change<br><br>Implementation - Increased<br><br>Maintenance-Increased<br><br>Reimbursement or financial implications for the practice – Increased<br><br>Efficiency-Increased<br><br>Meaningful engagement-Increased

CO-CREATE Flyer Update	The team will be adding Antigen testing kit availability to the CO-CREATE flyer and updating the flyer illustrations.	Planned	The format	Tailoring to individuals<br><br>Adding a component	AIM 2: COVID-19 Testing	CRC<br><br>Principle or Co-Principle Investigator(s)	Late Implementation (AIM 2)	Based on pragmatic/ practical considerations (i.e., is this the only way it could work?)<br><br>Based on feedback or suggestions	To increase the number or type of patients contacted (reach)<br><br>To enhance the impact or success of the intervention for all or important subgroups (effectiveness)<br><br>To deliver intervention more consistently; better for practice, patient flow or EHR (implementation)<br><br>To institutionalize or sustain the intervention (maintenance)	Number or type of patients engaged reached- Increased<br><br>Effectiveness- Increased<br><br>Participation of teams or staff- No change<br><br>Implementation - No change<br><br>Maintenance- No change<br><br>Reimbursement or financial implications for the practice – Increased<br><br>Efficiency- No change<br><br>Meaningful engagement- No change
CO-CREATE Website	Team will be updating our website to include community events that CO-CREATE will be supporting.	Planned	The format	Adding a component	AIM 2: COVID-19 Testing	CRC	Late Implementation (AIM 2)	Based on our knowledge or experience of working with patients<br><br>Based on pragmatic/ practical considerations (i.e., is this the only way it could work?)	To increase the number or type of patients contacted (reach)<br><br>To enhance the impact or success of the intervention for all or important subgroups (effectiveness)<br><br>To deliver intervention more consistently; better for practice, patient flow or EHR (implementation)<br><br>For practical reasons (implementation)	Number or type of patients engaged reached- No change<br><br>Effectiveness- No change<br><br>Participation of teams or staff- No change<br><br>Implementation - No change<br><br>Maintenance- No change<br><br>Reimbursement or financial implications for the practice – No change<br><br>Efficiency- No change

										Meaningful engagement- No change
Change in Survey Distribution	Stopped using the tablets for survey entry at the beginning of the Omicron Surge. Benefit of this was less data lost and time efficiency.	Planned	The setting<br><br>The format	Removing a component	AIM 2: COVID-19 Testing	CRC	Late Implementation (AIM 2)	Based on pragmatic/ practical considerations (i.e., is this the only way it could work?)<br><br>Based on feedback or suggestions	To increase the number or type of patients contacted (reach)<br><br>To enhance the impact or success of the intervention for all or important subgroups (effectiveness)<br><br>To deliver intervention more consistently; better for practice, patient flow or EHR (implementation)<br><br>To institutionalize or sustain the intervention (maintenance)	Number or type of patients engaged reached- Increased<br><br>Effectiveness- Increased<br><br>Participation of teams or staff- No change<br><br>Implementation - No change<br><br>Maintenance- No change<br><br>Reimbursement or financial implications for the practice – No change<br><br>Efficiency- Increased<br><br>Meaningful engagement- No change

New Testing Hours	CO-CREATE has implemented new summer hours to help the team ease short staffed days and summer weather conditions. The new hours are 8am-1:30pm Monday- Friday.	Planned	The setting	Tailoring to individuals	AIM 2: COVID-19 Testing	Entire or most of team	Implementation (AIM 2)	Based on pragmatic/ practical considerations (i.e., is this the only way it could work?)<br><br>Based on feedback or suggestions	To enhance the impact or success of the intervention for all or important subgroups (effectiveness)<br><br>For practical reasons (implementation)<br><br>Other: To help with team burn out	Number or type of patients engaged reached- No change<br><br>Effectiveness- No change<br><br>Participation of teams or staff- No change<br><br>Implementation - Decreased<br><br>Maintenance- Increased<br><br>Reimbursement or financial implications for the practice – No change<br><br>Efficiency- No change<br><br>Meaningful engagement- No change
New Testing Days	CO-CREATE on-site team decided to remove Fridays from the testing schedule to help with team burnout, catching up with data entry and sustainability.	Planned	How the intervention/program is presented/delivered - how core components are operationalized	Tailoring to individuals	AIM 2: COVID-19 Testing	Entire or most of team	Implementation (AIM 2)	Based on pragmatic/ practical considerations (i.e., is this the only way it could work?)<br><br>Other: Sustainability	To enhance the impact or success of the intervention for all or important subgroups (effectiveness)<br><br>For practical reasons (implementation)<br><br>Other: sustainability	Number or type of patients engaged reached- No change<br><br>Effectiveness- No change<br><br>Participation of teams or staff- No change<br><br>Implementation - Decreased<br><br>Maintenance- Increased<br><br>Reimbursement or financial implications for the practice – No change<br><br>Efficiency- No change

										Meaningful engagement- No change
Antigen Testing/Schedule	To help provide effective testing experience and help team manage short-staffed days antigen testing is offered Mon-Thur. from 8am-11am. This information has been communicated with UCSD providers and CO-CREATE patients.	Planned	How the intervention/program is presented/delivered - how core components are operationalized	Tailoring to individuals<br><br>Removing a component	AIM 2: COVID-19 Testing	Entire or most of team	Late Implementation (AIM 2)	Based on pragmatic/ practical considerations (i.e., is this the only way it could work?)<br><br>Based on feedback or suggestions	To enhance the impact or success of the intervention for all or important subgroups (effectiveness)<br><br>To enhance the impact or success of the intervention for all or important subgroups (effectiveness)	Number or type of patients engaged reached- No change<br><br>Effectiveness- No change<br><br>Participation of teams or staff- No change<br><br>Implementation - No change<br><br>Maintenance- No change<br><br>Reimbursement or financial implications for the practice – No change<br><br>Efficiency- No change<br><br>Meaningful engagement- No change

Verbal Consent	IRB amendment approval to have participants verbally consent and reconsent to study. Participants receive physical copy of consent to keep and team keeps marked copy.	Planned	How the intervention/ program is presented/delivered - how core components are operationalized	Substituting for a component	AIM 2: COVID-19 Testing	Entire or most of team	Implementation (AIM 2)	Based on our knowledge or experience of working with patients	To enhance the impact or success of the intervention for all or important subgroups (effectiveness)	Number or type of patients engaged reached- No change<br><br>Effectiveness- No change<br><br>Participation of teams or staff- No change<br><br>Implementation - No change<br><br>Maintenance- No change<br><br>Reimbursement or financial implications for the practice – Increased<br><br>Efficiency- Increased<br><br>Meaningful engagement- No change
Limit on Arm 1 visits	Approved IRB amendment that limits participants to two visits per week and a total of 55 visits a year under Arm 1.	Planned	How the intervention/program is presented/delivered - how core components are operationalized	Substituting for a component	AIM 2: COVID-19 Testing	CRC	Implementation (AIM 2)	Based on pragmatic/ practical considerations (i.e., is this the only way it could work?)	Other: To align with UCSD compensation tax policies and avoid participants from having to file tax forms	Number or type of patients engaged reached- No change<br><br>Effectiveness- No change<br><br>Participation of teams or staff- No change<br><br>Implementation - No change<br><br>Maintenance- No change<br><br>Reimbursement or financial implications for the practice – Increased<br><br>Efficiency- No change

										Meaningful engagement- No change
Decision Tree	IRB approved decision tree is used to determine what participants are eligible for an antigen tests. As part of the registration process, every participant is asked about their symptoms and whether they have tested positive in the last 90 days.	Planned	Personnel involved	Adding a component	AIM 2: COVID-19 Testing	Entire or most of team	Implementation (AIM 2)	Based on pragmatic/ practical considerations (i.e., is this the only way it could work?)	To increase the number or type of patients contacted (reach)<br><br>To deliver intervention more consistently; better for practice, patient flow or EHR (implementation	Number or type of patients engaged reached- No change<br><br>Effectiveness- No change<br><br>Participation of teams or staff- No change<br><br>Implementation - No change<br><br>Maintenance- No change<br><br>Reimbursement or financial implications for the practice – No change<br><br>Efficiency- Increased<br><br>Meaningful engagement- No change

Traffic signal result template	Return of result template now includes a traffic signal image with the color corresponding to their result: green is negative, red is positive, and yellow is unsatisfactory and inconclusive	Planned	How the intervention/program is presented/delivered - how core components are operationalized	Extending a component	AIM 2: COVID-19 Testing	CRC	Implementation (AIM 2)	Based on our knowledge or experience of working with patients	To enhance the impact or success of the intervention for all or important subgroups (effectiveness)	Number or type of patients engaged reached- No change<br><br>Effectiveness- No change<br><br>Participation of teams or staff- No change<br><br>Implementation - Increased<br><br>Maintenance- No change<br><br>Reimbursement or financial implications for the practice – No change<br><br>Efficiency- No change<br><br>Meaningful engagement- No change
New SYH staff	SYH hired a new research assistant to help the CO-CREATE team.	Planned	Personnel involved	Other: Staff	AIM 2: COVID-19 Testing	CRC	Implementation (AIM 2)	Based on our knowledge or experience of working with patients	To increase the number or type of patients contacted (reach)	Number or type of patients engaged reached- No change<br><br>Effectiveness- No change<br><br>Participation of teams or staff- No change<br><br>Implementation - No change<br><br>Maintenance- No change<br><br>Reimbursement or financial implications for the practice – No change<br><br>Efficiency- No change

										Meaningful engagement- No change
On-site testing flow to include antigen testing	On-site there is now a designated staff member each day that conducts CDPH antigen testing. After registration, all eligible participants go to designated staff member for testing.	Planned	Personnel involved<br><br>The setting	Adding a component	AIM 2: COVID-19 Testing	SYH Research Staff	Implementation (AIM 2)	Based on pragmatic/ practical considerations (i.e., is this the only way it could work?)	To enhance the impact or success of the intervention for all or important subgroups (effectiveness)	Number or type of patients engaged reached- No change<br><br>Effectiveness- No change<br><br>Participation of teams or staff- No change<br><br>Implementation - No change<br><br>Maintenance- No change<br><br>Reimbursement or financial implications for the practice – No change<br><br>Efficiency- Increased<br><br>Meaningful engagement- No change

On-site set up	To alleviate the sun and heat, multiple canopies were added by SYH plant support. The canopies protect equipment, staff and participants from the sun	Planned	The setting	Other: the environment	AIM 2: COVID-19 Testing	Entire or most of team	Implementation (AIM 2)	Based on pragmatic/practical considerations (ex: is this the only way it could work?)	Other: To maintain safety of staff and equipment	Reimbursement or financial implications for the practice – Increased
Preferred name addition to redcap	REDCap testing detail form now has a preferred name section available for staff to add any other names participants prefer.	Planned	Other: Data collection	Tailoring to individuals	AIM 2: COVID-19 Testing	CRC<br><br>SYH Research Staff	Implementation (AIM 2)	Based on our knowledge or experience of working with patients	To enhance the impact or success of the intervention for all or important subgroups (effectiveness)	Number or type of patients engaged reached- No change<br><br>Effectiveness- No change<br><br>Participation of teams or staff- No change<br><br>Implementation - No change<br><br>Maintenance- No change<br><br>Reimbursement or financial implications for the practice – No change<br><br>Efficiency- No change<br><br>Meaningful engagement- Increased

New courier	Dropoff is now the currier for CO-CREATE. They deliver tests to EXCITE using new software that allows team to see the curriers location.	Planned	Personnel involved	Substituting a component	AIM 2: COVID-19 Testing	CRC<br><br>SYH Research Staff	Implementation (AIM 2)	Based on pragmatic/practical considerations (ex: is this the only way it could work?)	To institutionalize or sustain the intervention (maintenance)	Reimbursement or financial implications for the practice – Increased
Location Change	Testing moved from main clinic parking lot to the original MCHC CO-CREATE location.	Planned	The setting	Other:Location	AIM 2: COVID-19 Testing	SYH Research Staff<br><br>SYH Investigator(s)	Implementation (AIM 2)	Based on feedback or suggestions	To institutionalize or sustain the intervention (maintenance)	Number or type of patients engaged reached- No change<br><br>Effectiveness- No change<br><br>Participation of teams or staff- No change<br><br>Implementation - No change<br><br>Maintenance- No change<br><br>Reimbursement or financial implications for the practice – No change<br><br>Efficiency-No change<br><br>Meaningful engagement- No change

Student Volunteers to Interns	Our student volunteers promoted to paid interns as a way to increase staff members for CO-CREATE and as a response to growing demand on-site.	Planned	Personnel involved	Adding a component	AIM 2: COVID-19 Testing	Entire or most of team	Early Implementation (AIM 2)	Based on pragmatic/ practical considerations (i.e., is this the only way it could work?)	To make it possible to involve more teams, team members or staff (adoption)<br><br>For practical reasons (implementation)	Number or type of patients engaged reached- Increased<br><br>Effectiveness- Increased<br><br>Participation of teams or staff- Increased<br><br>Implementation - Increased<br><br>Maintenance- Increased<br><br>Reimbursement or financial implications for the practice – Increased<br><br>Efficiency- Increased<br><br>Meaningful engagement- No change
